# Protein Data Bank Japan: Celebrating our 20th anniversary during a global pandemic as the Asian hub of three dimensional macromolecular structural data

**DOI:** 10.1002/pro.4211

**Published:** 2021-10-27

**Authors:** Gert‐Jan Bekker, Masashi Yokochi, Hirofumi Suzuki, Yasuyo Ikegawa, Takeshi Iwata, Takahiro Kudou, Kei Yura, Toshimichi Fujiwara, Takeshi Kawabata, Genji Kurisu

**Affiliations:** ^1^ Institute for Protein Research Osaka University Suita Osaka Japan; ^2^ School of Advanced Science and Engineering Waseda University Shinjuku Tokyo Japan; ^3^ Graduate School of Humanities and Sciences, Ochanoizu University Bunkyo Tokyo Japan; ^4^ Protein Research Foundation Minoh Osaka Japan; ^5^ Graduate School of Frontier Biosciences Osaka University Suita Osaka Japan

**Keywords:** BMRB, COVID‐19, EMDB, PDB, protein structures

## Abstract

Protein Data Bank Japan (PDBj), a founding member of the worldwide Protein Data Bank (wwPDB) has accepted, processed and distributed experimentally determined biological macromolecular structures for 20 years. During that time, we have continuously made major improvements to our query search interface of PDBj Mine 2, the BMRBj web interface, and EM Navigator for PDB/BMRB/EMDB entries. PDBj also serves PDB‐related secondary database data, original web‐based modeling services such as Homology modeling of complex structure (HOMCOS), visualization services and utility tools, which we have continuously enhanced and expanded throughout the years. In addition, we have recently developed several unique archives, BSM‐Arc for computational structure models, and XRDa for raw X‐ray diffraction images, both of which promote open science in the structural biology community. During the COVID‐19 pandemic, PDBj has also started to provide feature pages for COVID‐19 related entries across all available archives at PDBj from raw experimental data and PDB structural data to computationally predicted models, while also providing COVID‐19 outreach content for high school students and teachers.

## 1 INTRODUCTION

Protein Data Bank Japan (PDBj, https://pdbj.org), has accepted and processed the three dimensional (3D) structure data of biological macromolecules from Asia since 2000. Since our inception, PDBj had processed data from Oceania as well, but since 2016, the worldwide Protein Data Bank (wwPDB, https://wwpdb.org), which was founded by RCSB PDB in the United States, PDBe in Europe and PDBj in Asia, reassigned the corresponding geographical regions of the three regional PDB data centers based on deposition statistics. This has resulted in PDBj focusing on Asian countries, which have seen a rapid increase in depositions in recent years, and the Middle‐East. In total, roughly 23% of all PDB entries had been processed by PDBj by the end of 2020. Although 2020 was a year of hardship that heralded a new chapter in the history of humankind, we also celebrated the 20th anniversary of our regional PDB activities in 2020. Here, we will describe a brief history of PDBj, updates to our original services and our regional outreach activities during the COVID‐19 pandemic.

### 1.1 History of PDB activities in Japan

**FIGURE 1:**
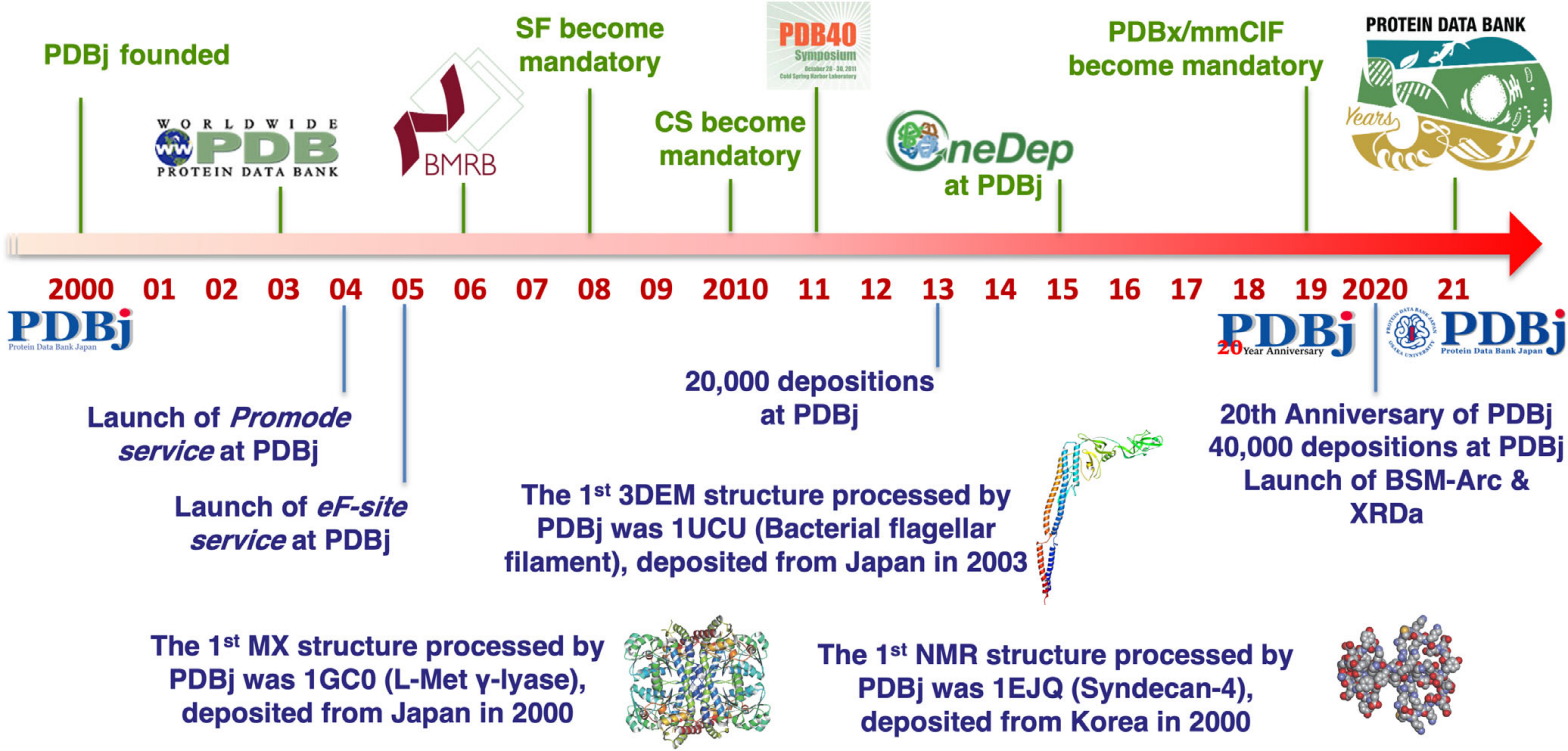
History of Protein Data Bank Japan over the past 20 years. Several milestones of PDBj are listed, in addition to the wwPDB's

Our regional PDB‐related activities have a much longer history, dating back to well before the establishment of PDBj. Our pre‐PDBj activities started in 1971, with the first protein structure from Asia, the X‐ray structure of bonito cytochrome *c*, determined at 4 Å resolution at the Institute for Protein Research (IPR), Osaka University.^1^ The subsequent atomic coordinates at 2.3 Å were deposited to the PDB in 1976 as the 21st entry of the PDB archive (PDB ID: 1cyc).^2^ Based on this early contribution as a depositor, IPR started a PDB data distribution service from 1979^3^ and kept serving the PDB data as a regional data center, initially by magnetic tape and later by CD‐ROM, until its official designation as a mirror site of Brookhaven PDB in 1998.^4^ In 2000, IPR formed PDBj with the support of JST (Japan Science and Technology Agency), and PDBj started local data annotation at Osaka with strong technical support from RCSB PDB (Figure 1). Τhe first MX entry processed by PDBj was the X‐ray structure of L‐methionine γ‐lyase (PDB ID: 1gc0), deposited on July 6th, 2000, from Japan.^5^ The first NMR entry processed by PDBj was the NMR structure of Syndecan‐4 (PDB ID: 1ejq), deposited in March 4th, 2000, from Korea.^6^ Finally, the first EM entry processed by PDBj was the bacterial flagellar filament structure (PDB ID: 1ucu) deposited on April 4th, 2003, from Japan.^7^ In its first year, PDBj processed 157 entries, followed by 376 entries in 2001, and 602 entries in 2002. Since 2003, PDBj has collaborated globally on our data‐in activities as a founding member of the wwPDB.^8^ In addition to structural data, PDBj also accepts and processes raw NMR experimental data. The Biological Magnetic Resonance Data Bank Japan (BMRBj, formerly known as PDBj‐BMRB, https://bmrbj.pdbj.org) is a regional NMR data repository of biomolecules in close collaboration with BMRB at UConn Health,^9^ which was launched by us in 2002 to cope with the rapid growth of NMR structural data at that time in Japan. The first BMRB entry processed at PDBj was released in 2005 and since then, 10% of BMRB entries have been processed by BMRBj. Since 2001, we have provided our in‐house developed online Data‐out services freely and publicly to the scientific community through our website. Our original services include our developed secondary databases and new unique archives. We developed secondary databases such as eF‐site, molecular surfaces of functional sites, Promode Elastic, a database of protein dynamics calculated via normal mode analysis (NMA), and HOMCOS, a server for searching and modeling of 3D structure of complexes. Finally, we launched a new archive called the Biological Structural Model Archive, BSM‐Arc (https://bsma.pdbj.org) for raw data and structures obtain via computational approaches; and the X‐Ray Diffraction Archive, XRDa (https://xrda.pdbj.org) for raw diffraction image data.

### 1.2 Overview of PDBj tools and services

Over the past two decades, PDBj has developed many unique and powerful services and tools.^10^, ^11^ Our primary services revolve around the PDB data that we co‐maintain together with RCSB PDB and PDBe. We provide the official PDB core archive at https://ftp.pdbj.org/ and the versioned PDB data at https://pdb-versioned.pdbj.org/. We also co‐maintain the EMDB archive containing experimental 3DEM maps together with the EMDB team at EMBL‐EBI in Europe, who recently became an official member of the wwPDB. In addition, we also provide supplementary data for the PDB, secondary database data, and data from our unique archives BSM‐Arc and XRDa, which are all available via our data archive at https://data.pdbj.org/. Table 1 lists the major services that we have developed and currently maintain. To search and explore published PDB data, we have developed a search service called PDBj Mine, which can be used to quickly search PDB data, as well as to perform very complex search criteria and data analyses via our SQL interface. For each entry, individual pages exist that function as portals to analyze the structure in terms of both their metadata, as well as their 3D structure, and linking to both internal and external databases. For the EMDB, we have also developed the EM Navigator search service, which can be used to search EMDB entries similar to PDBj Mine for PDB data, with individual EMDB entry pages that provide in‐depth information about the structures. Finally, for NMR data, PDBj collaborates with the BMRB team in the USA for maintaining the BMRB archive as part of our BMRBj activities, which can be used to search and explore the BMRB archive similar to our PDBj Mine and EM Navigator services for the PDB and EMDB, respectively.

**TABLE 1:** PDBj services and tools with corresponding URLs

Service	URL
Search PDB (PDBj Mine)	pdbj.org/search/pdb‐filter
Chemie search	pdbj.org/chemie‐search
Search BMRB	bmrbj.pdbj.org
Sequence‐Navigator	pdbj.org/seq‐navi
EM Navigator	pdbj.org/emnavi
Omokage search	pdbj.org/omokage
wwPDB/RDF	rdf.wwpdb.org
jV: Graphic Viewer	pdbj.org/jv/
Molmil: WebGL Molecular Viewer	pdbj.org/molmil2/
Yorodumi	pdbj.org/emnavi/
NMRToolBox	bmrbj.pdbj.org/en/nmr_tool_box.html
gmfit	pdbj.org/gmfit/
CRNPRED	pdbj.org/crnpred/
HOMCOS	homcos.pdbj.org
eF‐site	pdbj.org/eF‐site/
eF‐seek	pdbj.org/eF‐seek/
eF‐surf	pdbj.org/eF‐surf/
ProMode Elastic	pdbj.org/promode‐elastic
Molecule of the Month	numon.pdbj.org/mom/
Games	numon.pdbj.org/games/
Papermodels	numon.pdbj.org/papermodel/
OneDep(Deposition to PDB, EMDB, or BMRB)	deposit‐pdbj.wwpdb.org/deposition
Format Conversion	mmcif.pdbj.org/converter/
PDBx/mmCIF editor	pdbj.org/cif‐editor/
EMPIAR‐PDBj	empiar.pdbj.org
BSM‐Arc	bsma.pdbj.org
XRDa	xrda.pdbj.org
COVID‐19 pages	pdbj.org/featured/covid‐19

In addition to these core tools, we have also developed additional tools and services. To enable sequence homology searches within the PDB, we provide the Sequence Navigator service, which enables searching the PDB for homologous structures given a query sequence. Similarly, for existing PDB entries, our Sequence Neighbor service enables searching for homologous structures and visualizing their superposed structures using our molecular viewer Molmil.^12^ With Chemie, we provide a search interface to the chemical compound dictionary data of the PDB. The search interface enables searching the compound library and similar to PDBj Mine for PDB entries, individual entry pages are also available, providing information about the chemical structure, visualization using Molmil and links to PDB entries that contain the chemical compound, with a similar interface also provided for PRD/BIRD (Biologically Interesting Molecule Reference Dictionary) entries. Molmil is a WebGL‐based molecular viewer launched in 2013 and is used throughout the PDBj website by various services and replaces our old Java‐based molecular viewer jV,^13^ which can no longer be used in modern web browsers. Molmil can also be used as a standalone viewer to load user‐provided structures, without requiring any installation (although an installable version called Molmil‐app is also available to enable shell‐based loading and headless processing). During the process of developing Molmil, we have also developed a new format called PDBx/mmJSON, which uses the same definitions (and dictionary) as PDBx/mmCIF, but encodes the data in a JSON format, which can be read by any modern programming language, without requiring any custom mmCIF parser. Separately, we have also developed an RDF service that provides the PDB data in an RDF format.

Recently, structure data by a set of 3D analysis methods using electron microscopy (3DEM), including single particle analysis, electron tomography and electron diffraction, are rapidly increasing. In 2007, we started our EM Navigator service,^14^ a website to explore 3DEM data in the EMDB and PDB. At that time, 3DEM methods were only a minor contributor to the PDB, and only few users had the skills to view and manipulate 3DEM data, such as a 3D density map. Typical 3DEM atomic models were difficult to view in web browsers, since some of them were too large to be included in a single PDB entry, while others were asymmetric units of assemblies corresponding to a higher order symmetry, such as an icosahedral one. To visualize EMDB map data and/or PDB model data without any special skills or software, we started to employ short movies to enable users to view the data from different orientations. In recent years, in addition to the improvements to the EM Navigator interface, the “Recently Published Data” area on the EM Navigator top page has been improved to accommodate the rapid increase in the number of entries released every week, while search results and statistics can also be downloaded in CSV, TSV, or JSON format. We have also started to provide additional services that enable structure‐based searches for EM data. The Omokage service is a shape similarity search service for 3D structures of macromolecules that compares the overall shape between registered structures and/or a user‐submitted one.^15^ The gmfit service also works on EM data and can be used to quickly fit 3D objects (either structures or density maps) using Gaussian mixture models.^16^

One of our oldest services is CRNPRED,^17^ which can be used to predict characteristics of a protein such as secondary structure, contact numbers, and residue‐wise contact orders from the amino acid sequence. While CRNPRED uses the amino acid sequence to predict structural properties, our HOMCOS service can be used to model the quaternary structure of proteins based on homology modeling.^18^ In addition, it can also be used to search for potential binding compounds given an amino acid sequence, or a set of binding proteins given a compound. Contrarily, our eF‐seek service can be used to find proteins that have similar binding sites based on the shape and electrostatic properties of a query pocket,^19^ using the surfaces and properties registered in our secondary database eF‐site,^20^ where such surfaces can also be generated from user provided data using our eF‐surf service. The Promode Elastic service is also a secondary database, but provides information with respect to predicted dynamics of a PDB entry (or specific chains), calculated via NMA.^21^

Besides services related to our data‐out activities, we also provide services regarding our data‐in activities. Foremost is OneDep, the unified deposition platform by the wwPDB, which each of the PDB regional partners (RCSB PDB, PDBe, and PDBj) uses to receive and process depositions from experimentalists,^2^ with our annotators processing the depositions in the Asian area. Experimental NMR data can be submitted through OneDep,^22^ BMRBdep, or SMSDep, depending on the size of the biological macromolecule and whether atomic coordinates are included or not, where OneDep now supports deposition in either NEF (NMR Exchange Format) or NMR‐STAR format.^23^, ^24^ To assist with the deposition, we provide two additional services. First is a format conversion service where we use the MAXIT software to convert mmCIF to PDB files and vice versa. Second one is a CIF editor, which can be used to load and modify existing mmCIF files using a GUI and can be used directly in the web browser without any installation.^25^

Over the past couple of years, we have also introduced several new archives. The EMPIAR^26^ archive was initially developed by EMBL‐EBI and we now host a mirror site at PDBj (https://empiar.pdbj.org). In addition, we assist with depositions from Asia, where depositors can upload their data to EMPIAR‐PDBj, or send their hard disk drives via postal mail or a courier service to us and we will then process their data. We have also developed two unique new archives. The first is BSM‐Arc, which accepts structure models and raw data obtained via computational methods such as molecular dynamics, homology modeling, or deep learning based methods.^27^ Finally, our most recent archive is XRDa, which accepts raw X‐ray diffraction images for existing and new PDB entries. Thus, PDBj collects experimental structural models via OneDep, raw NMR data via BMRBj, raw EM image data via EMPIAR‐PDBj, raw X‐ray diffraction images via XRDa and finally computational structural models and raw data via BSM‐Arc, and is thus the only wwPDB partner that collects raw data for all experimental types and from computational sources.

In recent years, a number of improvements have been made to the PDBj interface, services and tools. We have added some new functionality to the Mine Search interface, added additional data to the Mine 2 relational database (RDB) and we have made extensive improvements to the integration of 3D structural data with our website. We have also introduced several new tools for modifying mmCIF files and local (and headless) visualization of 3D structural data using Molmil. Finally, we have made several improvements to our HOMCOS server for modeling 3D structures of proteins either in isolation or in complex with other biomolecules.

### 1.3 Updated query search interface for the PDB

**FIGURE 2:**
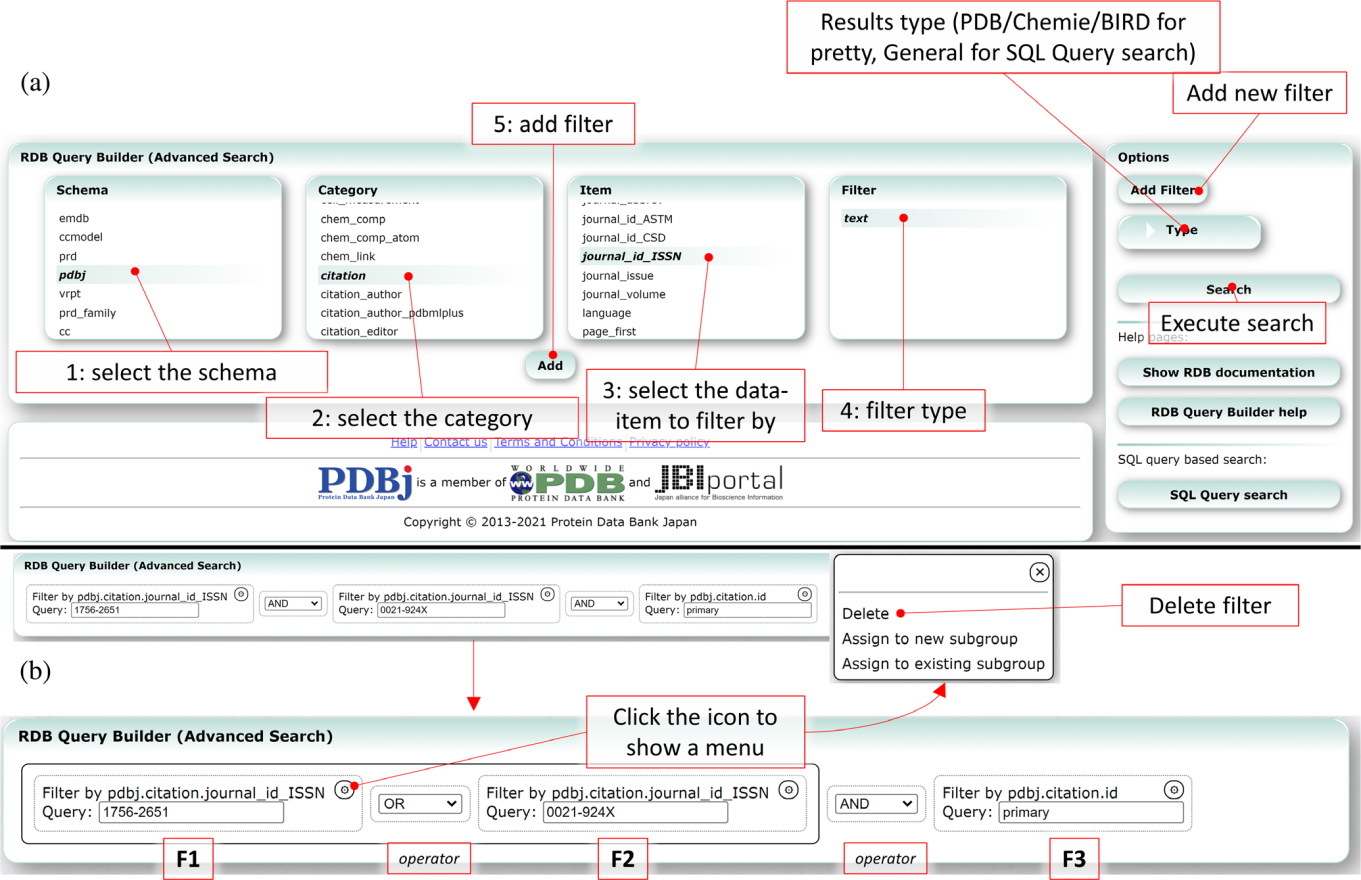
The new RDB Query Builder service (https://pdbj.org/rdb/build). The service can be used to perform advanced search queries to search the PDB using any of the registered metadata. (a) First, a user selects the schema to search against (Step 1). Then, in the category menu (Step 2) the available categories (or SQL tables) for the selected schema are listed. Upon selecting a category, the corresponding data items (or SQL columns) for the selected table are listed (Step 3). Finally (Step 4), a filter needs to be selected, which depends on the type of the selected data item. Clicking on the “Add” button adds the new filter (Step 5), and the user can then enter the query parameter(s). (b) Composite filters can be constructed by clicking the options menu of each individual filter to create new subgroups or add filters to existing subgroups. These groupings correspond to parenthesis used in the raw query, so that together with AND and OR operators, composite filters can be produced. In the example, the filter “(F1 OR F2) AND F3” is used, where each individual filter is created via the description in (a), followed by grouping the individual filters together via the options menu to generate the composite filter

We have made some reorganizations to our search interface, by merging some existing functionality and introducing a new graphical interface for our SQL Search interface. Previously, we offered an Advanced Search service that enabled searching based on a limited set of core criteria, but since it was not very advanced in comparison to our SQL search, we recently discontinued the service, although the functionality itself has been integrated in our regular Quick Search service. On the other hand, our SQL search service is extremely powerful and has enabled users to perform very specific search queries, but this powerful functionality is like a double‐edged sword. To effectively use our SQL search, users need to know both the SQL syntax and the data‐structure of mmCIF files, as the Mine 2 RDB follows a scheme similar to the mmCIF dictionary. This has long prevented most users from effective use of our SQL service, and has mostly been used by power users. To make the SQL search interface more accessible to regular users, we have designed a basic interface around the RDB that enables users to use advanced filters to search the PDB, as well as the Chemical Component Dictionary (CCD, that is, the data used by Chemie), PDB‐CSD integration data (ccmodel), PRD, SIFTS^28^ and miscellaneous meta data generated by us. However, making an interface that integrates all the functional capabilities of SQL would result in an unwieldy interface, one even more difficult to use than the SQL interface. For that reason, we have only opted to design an interface that enables easy filtering of PDB entries, similar to the basic filtering functionality of our Quick Search function, except that this filtering can be used on all metadata available in the RDB and some more complex filtering queries can be performed. The basic interface of the RDB Query Builder is shown in Figure 2, where via a basic interface (Figure 2a) a user can select the data source (e.g., PDB, chem_comp, etc.), an mmCIF category (a table within the RDB), an mmCIF data name (a column in the selected table), and a search type (e.g., a specific value or a range in case of a number). Here, we also offer documentation of the data sources available, categories and data names available with the RDB, to assist users unfamiliar with the mmCIF data structure to use the Mine 2 RDB. While the filtering functionality of the Quick Search functionality can only perform so‐called AND queries (i.e., only entries that satisfy all filters would match), the RDB Query Builder enables OR queries (i.e., it accepts entries that matches either filter) and subqueries, to enable very specific filtering (Figure 2b). The RDB Query Builder uses the specifications set by the user to generate an SQL query, which is then used to search the RDB, where the entries are then shown using the same interface as our Quick Search. In case nondefault data is requested, or filtering not compatible with the RDB Query Builder needs to be performed, a standard SQL Search can also be performed with the generated query, from which any further modifications can be made manually. In this case, the RDB Query Builder enables users (especially those unfamiliar with the mmCIF data structure) to prepare the initial query and then manually modify it to extract the data they need. The new RDB Query Builder service helps bridge the gap between our Quick Search service and our SQL Search service, making it easier for users to find the data they are looking for.

In addition to the introduction of the new RDB Query Builder, we have also revamped our data retrieval system. Previously, we offered an XPath service and a CIF Query service. These services enabled users to fetch specific data (either in XML or mmCIF/mmJSON format) for a single entry using either an XPath query, or by selecting a category to select the data for. In addition, we previously had a basic data retrieval system for the Quick Search data that could be used to fetch a limited set of data as a CSV/TSV/JSON file. These individual services have now been discontinued and have been replaced with a revamped data retrieval system. For all matches obtained by our PDB Quick Search and our RDB Query Builder services, it is now possible to obtain any and all meta data stored in the Mine 2 RDB. The data can be retrieved in mmJSON, mmCIF, row‐major JSON, column‐major JSON, CSV, or TSV formatted files. Similar to the data retrieval service, we also offer a batch download service for all matching entries. For all matching entries, files in mmJSON (all‐atom, header‐only, pdbjplus‐only), mmCIF, PDBML (all‐atom, header‐only), RDF, structure factor, and the legacy PDB flatfile format can be downloaded. The system splits up all the files into multiple batches (based on the number of files and their filesize) of zip files, to enable users to download a large set of files in a single or a small set of zipped files.

### 1.4 Improvements to PDB entry pages and 3D visualization

We have also made some improvements to individual PDB entry pages. Links to matching entries in our BSM‐Arc and XRDa archives have been added, along with links to for example, BRMBj and EM Navigator. If a higher resolution entry for the first entity is available, a link is also added to that structure, to help guide users towards potentially higher quality structures. Our sequence viewer functionality has also undergone a slight update, with improved visualization and annotation, and a new SNFG based visualization for branched molecules (glycans), where Molmil now also has support for 3D‐SNFG representation of glycans. Our Sequence Neighbor interface has also been improved, where users can now select any chain (instead of just a representative one for each entity) for either protein or DNA/RNA entities. The result interface has also been renewed, where the query structure can be superposed with any hit (instead of just a single representative from structures with the same sequence), to enable users to compare the structures that users are interested in. Finally, we have made considerable changes to our data distribution. We have prepared a new data archive (https://data.pdbj.org) that contains all PDB and EMDB data shared between the wwPDB partners, PDB versioning data, the PDBj generated data, the web‐data for PDBj's secondary databases eF‐site and Promode Elastic, EDMap data and the data of our new BSM‐Arc and XRDa archives. Our download and visualization functionality make use of the new data archive, but users can also download files manually (or automatically via scripts) using https (e.g., via wget/curl, including nongzipped files by omitting the .gz extension), ftp or the rsync protocol. We have added download links for files in the mmJSON format (all‐atom, metadata only and pdbjplus‐only), where visualization is handled by our CIF tree viewer functionality. These data are also used by our mine2updater application (https://gitlab.com/pdbjapan/mine2updater), which users can use to maintain their local installation of the Mine 2 RDB. Simultaneously, we have retired our old PDBMLplus format, as the same content is stored in both the mmJSON pdbjplus files (available from our data archive) and the Mine 2 RDB (available via our REST services).

To view the 3D structures, our PDB entry pages have long employed web‐based molecular viewers to quickly and easily visualize biomolecular structures. Over the past decade, there have been some large changes in the capabilities of web browsers. We used the Java based molecular viewers jV and Jmol for a long time on our website, and in 2013 we introduced our WebGL based molecular viewer Molmil. However, due to the deprecation of Java for web browsers, jV and Jmol have effectively become unusable as web‐based viewers. For this reason, we recently discontinued using the jV and Jmol viewers on the PDB entry pages and focused completely on Molmil instead. This enabled us to overhaul the interface, which had been shared between all viewers. As Molmil can also be used in standalone mode and is completely programmable via commands set in the URL, the new interface simply uses Molmil's standalone mode, where we added a new interface for the various PDBj services. Besides the standard Asymmetric Unit and Biological Unit visualization, visualization of eF‐site entries, electron density maps via our EDMap service and superposition of molecules for our Sequence Neighbor service have all been implemented in this new interface. Figure S1 shows our new interface for Molmil. Via the style menu, different visualization modes for the entry can be selected. In the past, our EDMap service made use of our own generated density maps based on the structure factors, but this now has been standardized by the wwPDB, where 2mfo‐Dfc and mfo‐Dfc maps are generated with the validation reports. Our EDMap service makes use of the standardized map data to generate the maps that are visualized by Molmil. Molmil is also being used by Promode Elastic, which recently has also undergone a major overhaul, integrating Molmil directly on the entry page, with a more modern and responsive interface and with fully interactive graphs (Figure S2).

Molmil itself has also undergone some improvements, including additional functionality added to enable superposition of structures, rendering of 3D‐SNFG, additional Pymol command support, and many minor improvements, where the source code is available at our repository (https://gitlab.com/pdbjapan/molmil). Molmil has also been integrated into BSM‐Arc, where Molmil scripts (.mjs files) can be used to load structures and apply custom styling, for example, to match figures included in scientific papers. Although Molmil has been able to load user files by drag‐and‐dropping them from the local HDD into Molmil, direct integration within the user's operating system (e.g., to load files via the shell or by double‐clicking on them) had been lacking. Recently, we introduced molmil‐app (https://gitlab.com/pdbjapan/molmil-app), which enables users to run Molmil as local application. The biggest advantage of Molmil is its automatic generation of images or movies, as Molmil can load files from the local hard drive and directly save them as PNG images or MP4 movies without user intervention, and this can even be done in headless mode (i.e., without a visible window and e.g., on a server). Combined with Molmil scripts, this allows for automatic and reproducible generation of images, greatly simplifying the process of creating images for scientific publications, where the scripts and data could afterwards also be submitted to BSM‐Arc, to make an interactive version of the images/movies publicly available.

### 1.5 Web‐based mmCIF editing tool

**FIGURE 3:**
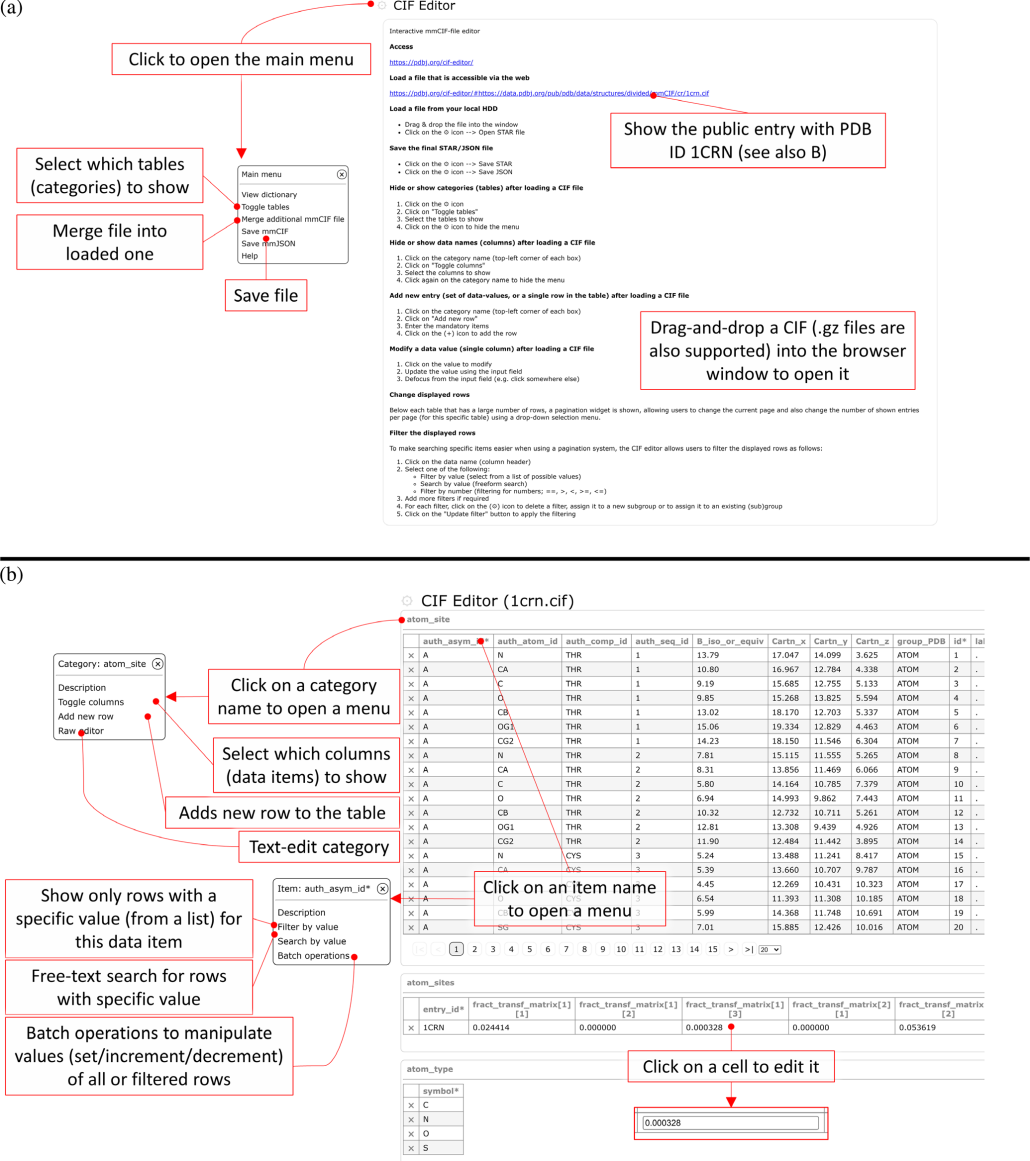
Overview of our CIF Editor. The editor can be used to load and modify STAR‐formatted files, including mmCIF, NMR‐STAR, and BSMA‐STAR. Files can be drag‐and‐dropped into the browser window after opening the page (https://pdbj.org/cif‐editor/) in panel (a), which will automatically load the file and check the entry against the corresponding dictionary (if configured in the input file, otherwise it will prompt the user to supply a dictionary). (b) Example using the public PDB entry with PDB ID 1crn. Upon loading a file or entry, the categories are represented as a table, where clicking on a cell will make it editable. The editor also has functionality to filter the rows and subsequently perform batch operations (e.g., to change the chain or residue ID) of the filtered rows. Finally, the data can be saved to a file (either as a STAR or JSON file in accordance with the dictionary) to the user's local hard drive

We recently introduced CIF editor,^25^ and the original announcement was in Japanese, hence we will re‐introduce it shortly here. To help depositors get used to the mmCIF format, we have created a new mmCIF editor, which is available at https://pdbj.org/cif-editor (Figure 3a), and due to its generalized implementation, the CIF editor is also used by BSM‐Arc and XRDa to modify metadata during deposition. Similar to Molmil, it runs inside a web browser and does not require any installation, ensuring that users will always use the most recent version, without having to wait to install an update before every use. The editor supports two modes; a UI based mode and a manual mode that allows users to directly edit the raw mmCIF data. Figure 3b shows an example of what a PDB entry loaded in our editor looks like. By default, the editor operates in this UI mode, which is primarily intended to help users unfamiliar with the mmCIF format along, while maintaining the data's integrity. Like Molmil, the mmCIF editor can also load local files from the hard drive via either the main menu or a drag‐and‐drop procedure. After editing the entry, the modified data can be saved back to an mmCIF file or to an mmJSON file from the main menu. The UI shows each category in a table reminiscent of PDBj's CIF tree viewer for mmCIF/mmJSON files, but allows users to edit the content. The category name functions as a button that pops up a menu to enable users to perform various operations on the selected category, such as adding new rows or toggling the visibility of columns (data‐names). Similar to the category name, each data‐name (column header) inside the table also functions as a button to show another menu that enables users to perform operations specific on the given data‐name, such as filtering and batch modifications. Individual values can be edited by clicking on them to show an edit field, while rows can be deleted either individually or via batch operations available from the category menu. Below each table is an interface to browse through subsections of a table and users can change the number of simultaneously displayed rows. While modifying or adding new data, the editor will ensure that any new data is compliant with the mmCIF dictionary. To enable users familiar with the mmCIF format to manually edit the content in a similar way as they might have done with the old flat file PDB format, the editor also supports a RAW editing mode. After switching to the RAW editing mode, users can freely edit the mmCIF data manually, after which the editor will re‐assimilate the modified content while validating it against the mmCIF dictionary. Finally, the data can be saved to the local hard drive via an option in the main menu.

### 1.6 Integrating KEGG chemical compounds in HOMCOS to enable template‐based docking

The HOMCOS server has been available since 2016 for both searching and modeling 3D complexes, using template‐based modeling.^18^ The HOMCOS server cannot only predict protein–protein complexes, but also protein‐compound complexes. HOMCOS internally uses BLAST^29^ and dkcombu^30^, ^31^ to search template proteins and chemical compounds, respectively, fkcombu^32^ to fit compounds to a template and generates a script for Modeller^33^ to enable user to perform detailed modeling of sidechains and loops. Since 2016, we have added several new functions to the HOMCOS server. To enable a rapid response to users' query sequences, we prepare precalculated results of BLAST and PSI‐BLAST^29^ for the latest Swiss‐Prot entries^34^ of several popular model organisms (*Escherichia coli*, human, mouse, and rat) in advance. When a user inputs one of these precalculated Swiss‐Prot identifiers as a query (e.g., CAC1C_HUMAN or NRX1A_HUMAN), the HOMCOS server can quickly return the search results. For these precalculated sequences, intrinsically disorder regions are also predicted by DISOPRED 3.1,^35^ to show for which regions a rigid 3D structure can be modeled, as shown in Figure S3.

**FIGURE 4:**
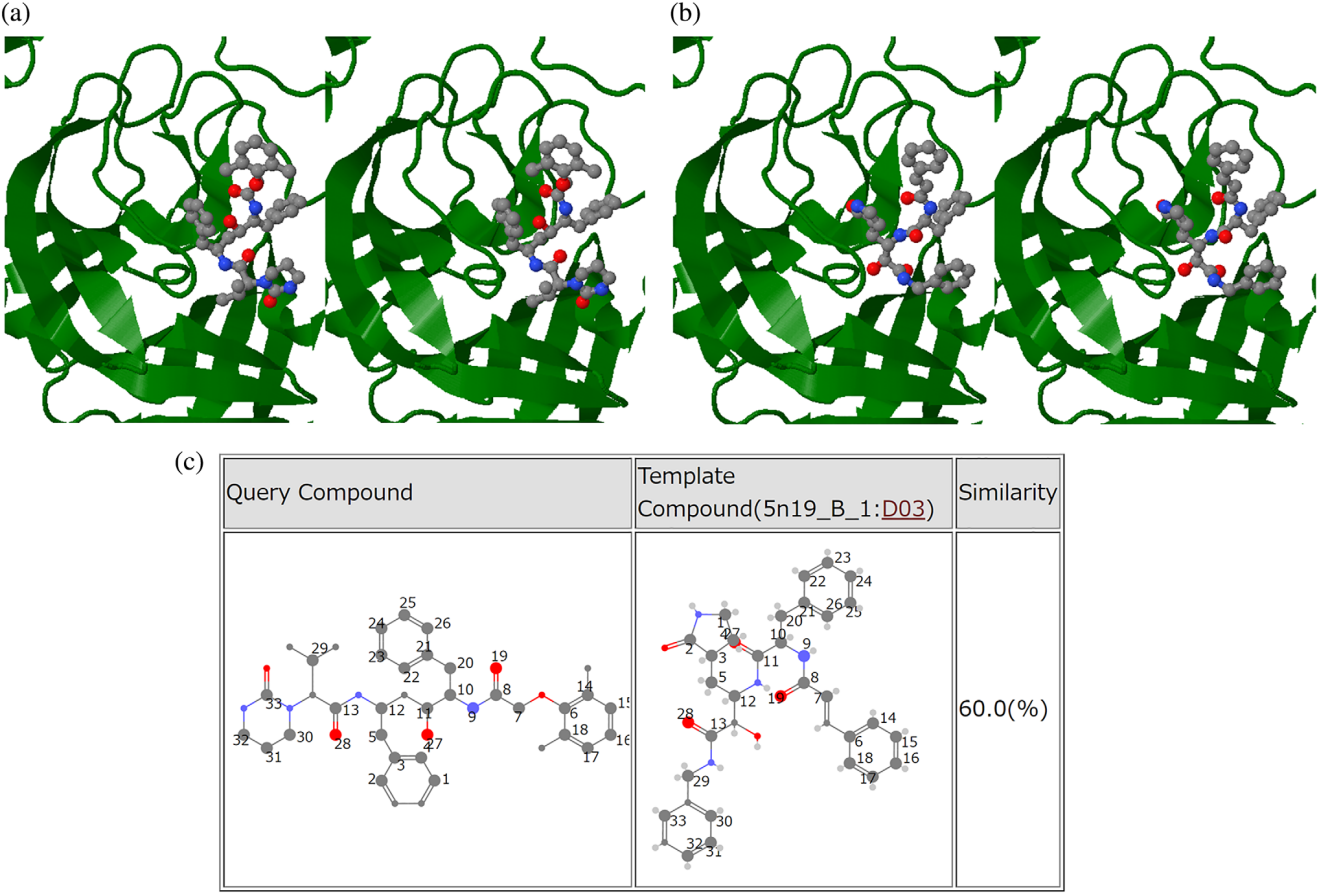
An example of modeled 3D complex structures of COVID‐19 related molecules by HOMCOS. (a) Template‐based docking model of Lopinavir (D01425) on the SARS coronavirus main protease in stereo view. (b) Template 3D structure (PDB ID: 5n19) of SARS coronavirus main protease and inhibitor (PDB comp_id: D03) in stereo view. (c) Atom‐matching pairs between Lopinavir (D01425) and inhibitor (PDB ID: 5n19, comp_id: D03). The matches are calculated using TD‐MCS *θ* = 1 by the KCOMBU program

To improve models involving compounds, we have integrated KEGG compounds into HOMCOS. More specifically, we now store generated 3D conformations of KEGG compounds^36^ on the server. Although template‐based 3D docking of chemical compound can be useful, the proper initial 3D conformation of the target compound is necessary for docking when the user does not provide a 3D conformation, for example, a SMILES string or a 2D structure sketch. Initial 3D structures from KEGG_COMPOUND and KEGG_DRUG are generated using Open Babel,^37^ after which we iterate between generating conformations using fkcombu^30^ and subsequently relaxing them using Open Babel^37^ until the chirality of the conformation matches the chiral description in KEGG, where the calculation procedure is described in more detail in Section S1 with statistics of the generated chemical compounds shown in Tables S1 and S2. Using this procedure, most of the generated 3D conformation have a consistent chirality to the KEGG compound, where the generated 3D conformations of the KEGG compounds are publicly available from the HOMCOS server (https://homcos.pdbj.org/cgi-bin/download_kegg.cgi). Finally, we have precalculated the chemical similarity between these KEGG compounds and the PDB compounds (CCD) using dkcombu,^30^, ^31^ where the statistics of the similarities between the two are shown in Tables S3 and S4. Here, about 40% of KEGG compounds do not have identical compounds in PDB, but have similar compounds with Tanimoto index of more than 0.7. Our previous study reported that if the reference and target compounds have more than 70% chemical similarity, then the average RMSD of the 3D conformation would be less than 2.0 Å.^32^ For such compounds, the HOMCOS server can quickly model accurate 3D conformations of protein‐compound complexes, just by submitting a KEGG identifier as the query, with an example of the modeling shown in Figure 4.

### 1.7 PDBj's outreach activities during a global pandemic

**FIGURE 5:**
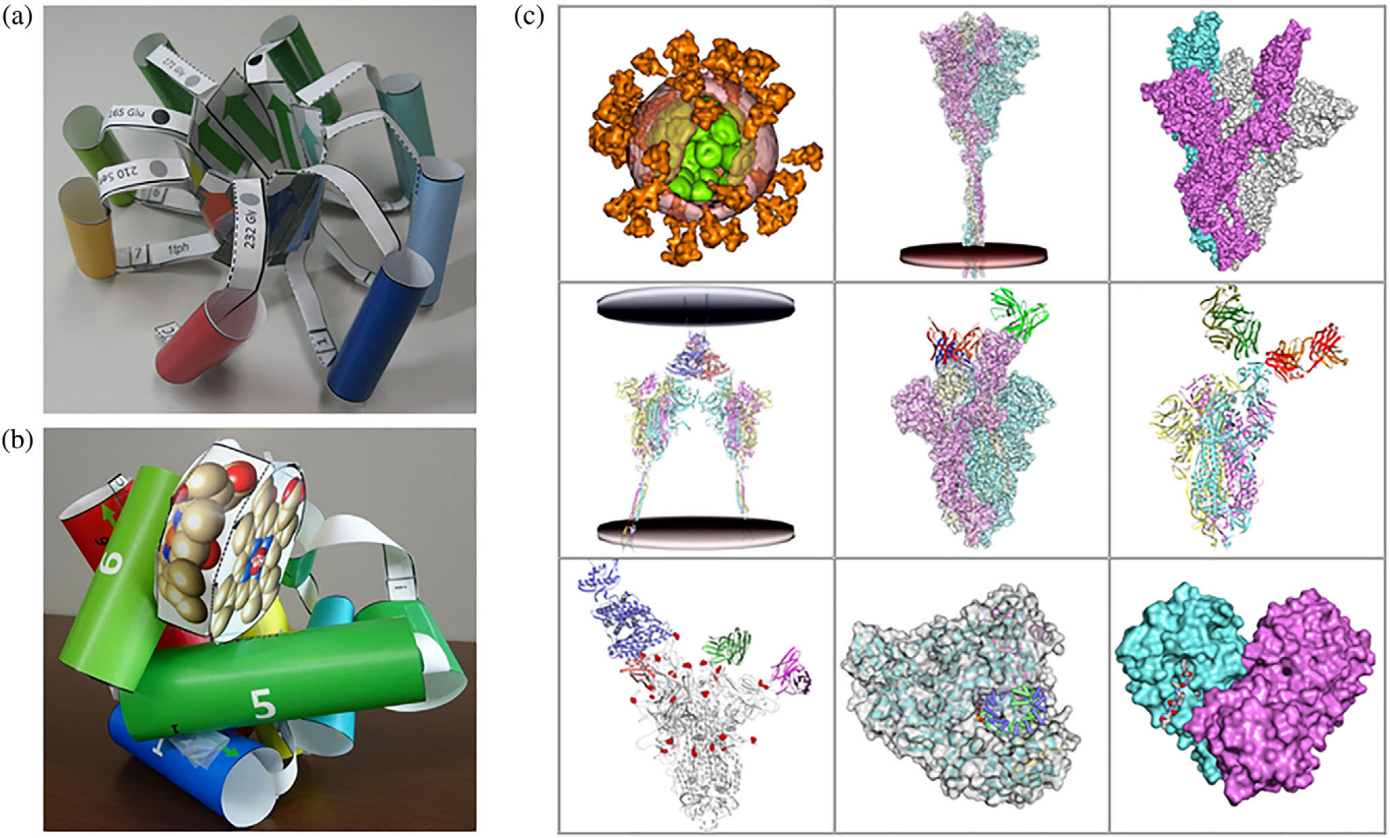
Educational materials for PDBj's outreach activities. (a) A papercraft model of a TIM barrel molecule. (b) A papercraft model of a myoglobin molecule. (c) Our educational page explaining the molecular mechanism of the coronavirus by employing 3D structures called “Shapes of proteins for living things: Molecules of COVID‐19”

PDBj Numon (https://numon.pdbj.org/, where “numon” means beginner's course in Japanese) is part of our outreach activities and includes educational resources to help students and nonspecialists to learn the 3D structures of biomolecules. Prepandemic, we would attend science fairs and use our Numon site to demonstrate the importance of proteins and the effect of their 3D structure. For children (and their parents), we provided papercraft models of biomolecules, which they could use to make a physical representation of the molecules either on‐site or at home. Since the start of the pandemic, these events have now moved online. As the papercraft files are available in PDF format, users can directly download the files from our website, print them and make the 3D models at home. Although most of the models are based on those provided by RCSB PDB, we have also designed several original paper models, such as for a TIM barrel, a GPCR molecule and myoglobin molecule, as shown in Figure 5a/b. Using Molmil as a base, we have also designed a VR environment where different molecules of public interest are placed in a virtual environment surrounding the user to explore the shape and relative size of the molecules. Using inexpensive VR goggles and a smartphone, users can also experience our VR environment at their own home. Similarly, our Yorodumi Prime service introduces biomolecules to the general public and displays an interactive stereo view using Molmil that can also be viewed at home using inexpensive red‐cyan anaglyph glasses. Finally, for our Molecule of the Month service, we provide Japanese translations from the original English articles part of the RCSB PDB's PDB‐101 (https://pdb101.rcsb.org/motm/).

**FIGURE 6:**
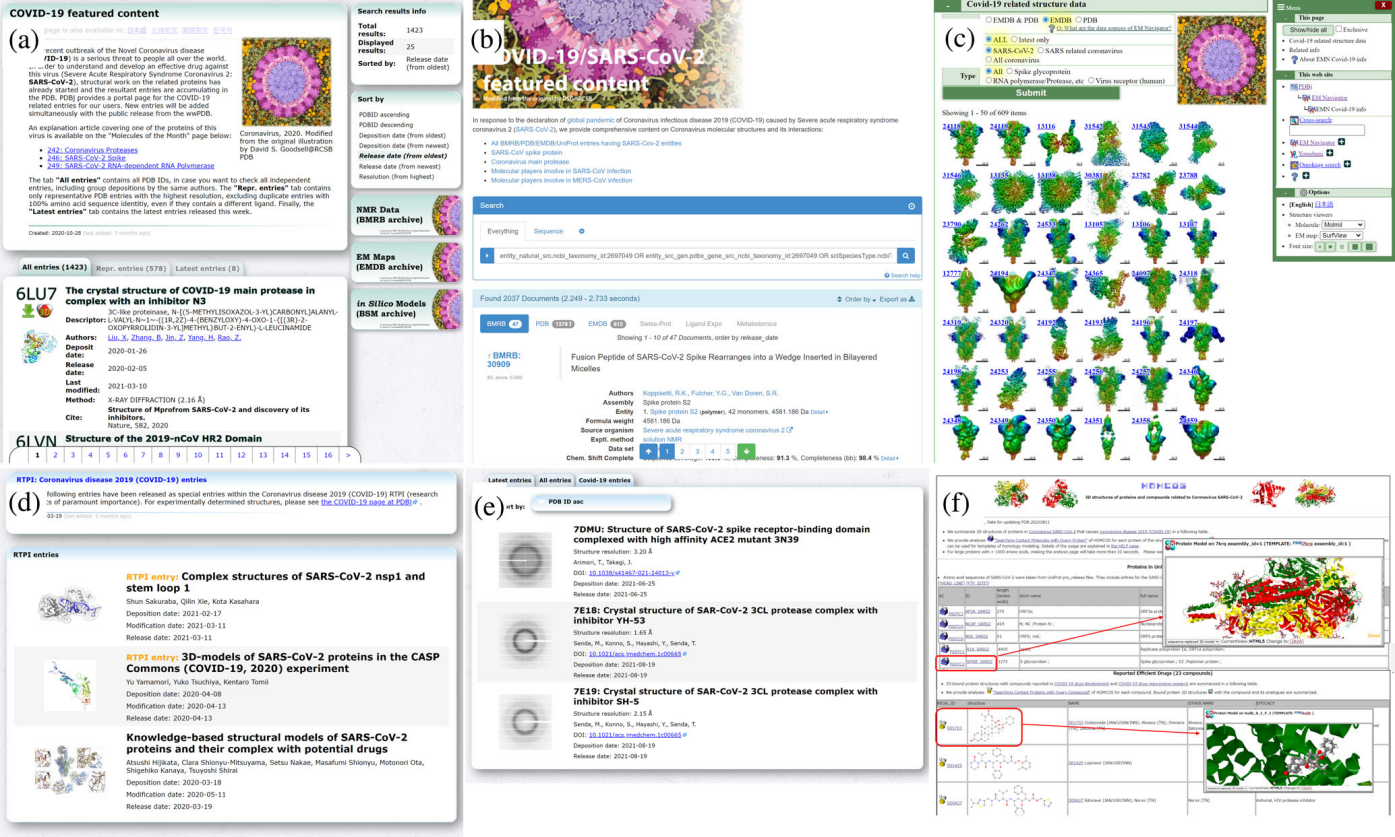
Overview of the various COVID‐19 pages provided by PDBj for various archives and services. (a) The main COVID‐19 page that lists the PDB entries (all entries, representative entries based on their sequence, this week's new entries). (b) COVID‐19 page for BMRB entries. (c) COVID‐19 page for EMDB entries. (d) COVID‐19 page for BSM‐Arc entries. (e) COVID‐19 page for XRDa entries. (f) COVID‐19 page by HOMCOS

To help the community get a better overview of the available entries related to COVID‐19, we have developed several feature pages that list all pertinent structural information related to COVID‐19 (Figure 6). The top page (https://pdbj.org/featured/covid-19) includes a list of all COVID‐19 PDB entries, a list of representative PDB entries (where duplicate structures are omitted) and a list of COVID‐19 PDB entries released that week. In addition, we also provide links to our other archives that each have their own COVID‐19 feature pages for EM Navigator, BMRBj and BSM‐Arc from this page. Using HOMCOS, we also provide an overview of known SARS‐CoV‐2 protein structures in UniProt, Genpept, and a list of existing drugs that are being investigated for their efficacy against COVID‐19 (https://homcos.pdbj.org/cgi-bin/sars_cov_2.cgi?LANG=en). The page (Figures 6f and S5) provides precalculated results of the HOMCOS service “Searching Contact Molecules for Query Proteins” for protein sequences related to SARS‐CoV‐2 (Figure S3), and those of the HOMCOS service “Searching Contact Proteins for Query Compound” for chemical compounds related to SARS‐CoV‐2 (Figure S4). The page shown in Figure S5 contains analyses not only of SARS‐CoV‐2 (41 proteins), but also SARS‐CoV (15 proteins) and human proteins (16 proteins) related to the virus. It also includes 23 potential drugs that have previously been approved for non‐COVID‐19 applications, taken from KEGG_DRUG. The details of the data preparation are described in Section S2. An example of the modeled 3D complex structure of Lopinavir is shown in Figure 4. The compound Lopinavir (D01425) is a protease inhibitor approved for treating HIV, but there is preliminary evidence of activity against the SARS and MERS.^38^ From the webpage, we confirmed that there was no reported 3D structure of Lopinavir and SARS‐CoV‐2 proteins, but we found one template complex structure (PDB ID 5n19)^39^ of the SARS main protease and an inhibitory compound similar to Lopinavir (Tanimoto index: 60%), as shown in Figures 4b,c and S4. Using this template, the HOMCOS server quickly modeled the complex 3D structure of Lopinavir on the SARS main protease (Figure 4a).

As part of our outreach activities, we have also created new webpages explaining the mechanism of the infection, mutations, and treatments of the coronavirus, by showing 3D structures of PDB and EMDB entries using Molmil (https://numon.pdbj.org/covid19/index.html.en), where an overview of the 3D models is shown in Figure 5c. Since 2020, more than 1,000 biomolecular structures related to COVID‐19 have been determined and registered in the PDB, and they have elucidated a detailed molecular mechanism of the disease. However, as these findings are often too technical for nonstructural biologists, we created these pages to help the general public, including students, to easily understand the structure of the coronavirus and the molecular mechanism of infection. To coincide with these pages, we have also prepared several movies to introduce the webpages and explain the molecular mechanism on our YouTube channel (https://www.youtube.com/user/PDBjmovie).

## 2 CONCLUSION

In the early days of the PDB, most entries were determined by simple X‐ray crystallography. However, in recent years, time‐resolved snapshot structures or large macromolecular complex structures using X‐ray Free Electron Laser, paramagnetism‐assisted NMR spectroscopy and 3DEM including sub‐tomogram averaging have been deposited to the PDB. Since these modern experimental techniques that provide PDB coordinates have become more integrated and difficult to understand by nonstructural biologists, it becomes increasingly important for users to easily find and use the appropriate entries from the wwPDB core archives. PDBj has developed and updated several original tools to help users to find/access/interoperate/reuse the PDB/BMRB/EMDB entries. Our COVID‐19 related outreach materials for nonstructural biologists is one example to show the importance of FAIR principles of the PDB archive. In parallel, PDBj has launched several new raw experimental and computational archives to promote open science and secure the regional experimental raw data deposition that is closely related to the PDB.

## CONFLICT OF INTEREST

The authors declare no potential conflict of interest.

## AUTHOR CONTRIBUTIONS

**Gert‐Jan Bekker:** Conceptualization (equal); data curation (lead); methodology (equal); software (lead); visualization (equal); writing – original draft (equal); writing – review and editing (equal). **Masashi Yokochi:** Software (equal); writing – original draft (equal). **Hirofumi Suzuki:** Data curation (equal); software (equal); writing – original draft (equal). **Yasuyo Ikegawa:** Data curation (equal). **Takeshi Iwata:** Data curation (equal). **Takahiro Kudou:** Visualization (equal); writing – original draft (equal). **Kei Yura:** Project administration (equal); supervision (equal). **Toshimichi Fujiwara:** Project administration (equal); supervision (equal). **Takeshi Kawabata:** Data curation (equal); methodology (equal); software (equal); supervision (equal); writing – original draft (equal); writing – review and editing (equal). **Genji Kurisu:** Conceptualization (equal); funding acquisition (lead); project administration (lead); supervision (lead); writing – original draft (equal); writing – review and editing (lead).

## Supporting information


**Appendix** S1: Supporting Information

**Appendix** S2: Supporting Information
