# Supplementary material for: Protein Data Bank Japan: Celebrating our 20th anniversary during a global pandemic as the Asian hub of three dimensional macromolecular structural data
Source: Protein Sci. 2021 Oct 27;31(1):173–86. doi: 10.1002/pro.4211 (PMC8740847; doi:10.1002/pro.4211)
Supplement: Supplementary file 1 — Appendix S1: Supporting Information [file PRO-31-173-s001.pdf]

Supplementary Information

Protein Data Bank Japan: Celebrating our 20th anniversary during a global pandemic as the Asian hub of 3D macromolecular structural data

Gert-Jan Bekker, Masashi Yokochi, Hirofumi Suzuki, Yasuyo Ikegawa, Takeshi Iwata, Takahiro Kudo, Kei Yura, Toshimichi Fujiwara, Takeshi Kawabata, Genji Kurisu

---

Figure S5. A special WEB page for corona virus in the HOMCOS server (https://homcos.pdbj.org/cgi-bin/sars_cov_2.cgi). Analyses of proteins and drugs related to the corona virus are listed: 44 UniProt proteins(13 SARS-CoV-2 proteins, 13 SARS-CoV proteins and 16 human proteins related to the virus), 28 SARS-CoV-2 proteins in GenPept, and 23 potential and approved drug in KEGG_DRUG. For each molecule, similar proteins and compounds and its binding molecules in PDB are summarized.

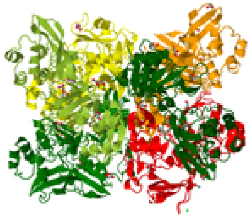

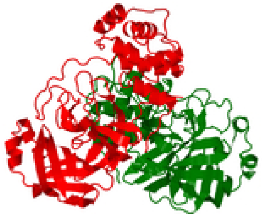

3D structures of proteins and compounds related to Coronavirus SARS-CoV-2

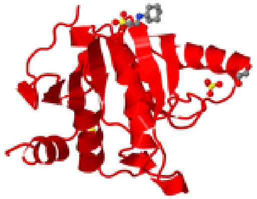

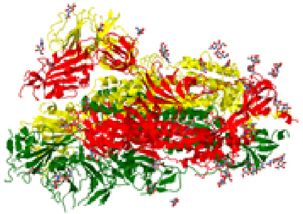

[Go to Japanese page]

Last Update:*2021/08/31*, Date for updating PDB:*20210811*

- We summarize 3D structures of proteins in Coronavirus SARS-CoV-2 that causes coronavirus disease 2019 (COVID-19) in a following table.
- We provide analyses "Searching Contact Molecules with Query Protein" of HOMCOS for each protein of the virus. Monomeric and bound 3D structures of the virus proteins and their homologs are summarized. Found 3D structures can be used for templates of homology modeling. Details of the usage are explained in the HELP page.
- For large proteins with > 1000 amino acids, making the analysis page will take more than 10 seconds. Please wait for a while.

Proteins in UniProt(44 entries)

- Amino acid sequences of SARS-CoV-2 were taken from UniProt pre_release files. They include entries for the SARS-CoV-2 virus (proteome UP000464024) and SARS-CoV virus (proteome UP000000354) and human target proteins. ([HEAD_LINE] [FTP_SITE])

AC	ID	length (amino acids)	Number of 3D homologues	short name	full name
	AP3A_SARS2	275	4	ORF3a;	ORF3a protein; Accessory protein 3a;Protein 3a;Protein

P0DTC3					U274;Protein X1;
P0DTC9	NCAP_SARS2	419	140	N; NC ;Protein N ;	Nucleoprotein ; Nucleocapsid protein ;
P0DTC6	NS6_SARS2	61	0	ORF6; ns6;	ORF6 protein; Accessory protein 6;Non-structural protein 6;Protein X3;
P0DTC7	NS7A_SARS2	121	4	ORF7a;	ORF7a protein; Accessory protein 7a;Protein U122;Protein X4;
P0DTD8	NS7B_SARS2	43	0	ORF7b;	ORF7b protein; Accessory protein 7b;
P0DTC8	NS8_SARS2	121	4	ORF8; ns8;	ORF8 protein; Non-structural protein 8;
P0DTD2	ORF9B_SARS2	97	12	ORF9b;	ORF9b protein; Accessory protein 9b;ORF-9b;Protein 9b;
P0DTD1	R1AB_SARS2	7096	1500	pp1ab;	Replicase polyprotein 1ab; ORF1ab polyprotein;
P0DTC1	R1A_SARS2	4405	1500	pp1a;	Replicase polyprotein 1a; ORF1a polyprotein;
P0DTC2	SPIKE_SARS2	1273	1473	S glycoprotein ;	Spike glycoprotein ; E2 ;Peplomer protein ;
P0DTC4	VEMP_SARS2	75	11	E;sM protein ;	Envelope small membrane protein ;
P0DTC5	VME1_SARS2	222	0	M;	Membrane protein ; E1 glycoprotein ;Matrix glycoprotein ;Membrane glycoprotein ;
P0DTD3	ORF9C_SARS2	73	0	ORF9c; ORF14;	ORF9c protein ; Uncharacterized protein 14;
P59632	AP3A_SARS	274	0		ORF3a protein; Accessory protein 3a;Protein 3a;Protein U274;Protein X1;
P59595	NCAP_SARS	422	0	NC ;Protein N ;	Nucleoprotein ; Nucleocapsid protein ;
P59633	NS3B_SARS	154	0	ns3b;	ORF3b protein; Accessory protein 3b;Non-structural protein 3b;Protein X2;

P59634	NS6_SARS	63	0	ns6;	ORF6 protein; Accessory protein 6;Non-structural protein 6;Protein X3;
P59635	NS7A_SARS	122	0		ORF7a protein; Accessory protein 7a;Protein U122;Protein X4;
Q7TFA1	NS7B_SARS	44	0	ns7b;	Protein non-structural 7b; Accessory protein 7b;
Q7TFA0	NS8A_SARS	39	0	ns8a;	ORF8a protein; Accessory protein 8a;Protein non-structural 8a;
Q80H93	NS8B_SARS	84	0	ns8b;	ORF8b protein; Accessory protein 8b;Non-structural protein 8b;
P59636	ORF9B_SARS	98	0		ORF9b protein; Accessory protein 9b;ORF-9b;Protein 9b;
P0C6X7	R1AB_SARS	7073	0	pp1ab;	Replicase polyprotein 1ab; ORF1ab polyprotein;
P0C6U8	R1A_SARS	4382	0	pp1a;	Replicase polyprotein 1a; ORF1a polyprotein;
P59594	SPIKE_SARS	1255	0	S glycoprotein ;	Spike glycoprotein ; E2 ;Peplomer protein ;
P59637	VEMP_SARS	76	0	E protein ;sM protein ;	Envelope small membrane protein ;
P59596	VME1_SARS	221	0	M protein ;	Membrane protein ; E1 glycoprotein ;Matrix glycoprotein ;Membrane glycoprotein ;
Q7TLC7	Y14_SARS	70	0		Uncharacterized protein 14;
Q9BYF1	ACE2_HUMAN	805	217	ACEH ;ACE-related carboxypeptidase;	Angiotensin-converting enzyme 2; Angiotensin-converting enzyme homolog ;Angiotensin-converting enzyme-related carboxypeptidase ;Metalloprotease MPROT15 {ECO:0000303|Ref.6};
Q10589	BST2_HUMAN	180	47	BST-2;	Bone marrow stromal antigen 2; HM1.24 antigen;Tetherin;
Q92499	DDX1_HUMAN	740	560	DBP-RB;	ATP-dependent RNA helicase DDX1; DEAD box protein 1;DEAD box protein retinoblastoma;
	FURIN_HUMAN	794	228	PACE;	Furin ; Dibasic-processing enzyme;Paired basic amino acid residue-

P09958					cleaving enzyme;
P52292	IMA1_HUMAN	529	344		Importin subunit alpha-1 ; Karyopherin subunit alpha-2;RAG cohort protein 1;SRP1-alpha;
P20701	ITAL_HUMAN	1170	329	LFA-1A;	Integrin alpha-L; CD11 antigen-like family member A;Leukocyte adhesion glycoprotein LFA-1 alpha chain;Leukocyte function-associated molecule 1 alpha chain;
Q8N3R9	PALS1_HUMAN	675	163		Protein PALS1 ; MAGUK p55 subfamily member 5;Membrane protein, palmitoylated 5 ;Protein associated with Lin-7 1 {ECO:0000303|PubMed:12527193, ECO:0000312|HGNC:HGNC:18669};
Q99623	PHB2_HUMAN	299	1		Prohibitin-2; B-cell receptor-associated protein BAP37;D-prohibitin;Repressor of estrogen receptor activity;
P35232	PHB_HUMAN	272	1		Prohibitin ;
O43765	SGTA_HUMAN	313	241	UBP;	Small glutamine-rich tetratricopeptide repeat-containing protein alpha; Alpha-SGT;Vpu-binding protein;
P84022	SMAD3_HUMAN	425	91	MAD homolog 3;Mad3;Mothers against DPP homolog 3;hMAD-3; SMAD 3;Smad3;hSMAD3;	Mothers against decapentaplegic homolog 3; JV15-2;SMAD family member 3;
O15393	TMPS2_HUMAN	492	1500		Transmembrane protease serine 2 ; Serine protease 10 ;
P15144	AMPN_HUMAN	967	335	AP-N;hAPN; AP-M;	Aminopeptidase N ; Alanyl aminopeptidase;Aminopeptidase M;Microsomal aminopeptidase;Myeloid plasma membrane glycoprotein CD13;gp150;
P08887	IL6RA_HUMAN	468	54	IL-6 receptor subunit alpha;IL-6R subunit alpha;IL-6R-alpha;IL-6RA; gp80;	Interleukin-6 receptor subunit alpha ; IL-6R 1;Membrane glycoprotein 80;
P40189	IL6RB_HUMAN	918	77	IL-6 receptor subunit beta;IL-6R subunit beta;IL-6R-beta;IL-6RB; gp130 ;	Interleukin-6 receptor subunit beta ; CDw130;Interleukin-6 signal transducer;Membrane glycoprotein 130;Oncostatin-M receptor subunit alpha;
	IL6_HUMAN	212	18	IL-6; BSF-2;CDF;IFN-beta-2;	Interleukin-6 ; B-cell stimulatory factor 2;CTL differentiation

P05231					factor;Hybridoma growth factor;Interferon beta-2;

Proteins in Genpept (28 proteins)

- Amino acid sequences of SARS-CoV-2 were taken from NCBI Reference Sequence. ([NC_045512]Severe acute respiratory syndrome coronavirus 2 isolate Wuhan-Hu-1, complete genome)

protein_id	length (amino acids)	Number of 3D homologues	product	gene	note
YP_009724389.1	7096	1500	2'-O-ribose methyltransferase	orf1ab	pp1ab; translated by -1 ribosomal frameshift
YP_009725297.1	180	19	leader protein		nsp1; produced by both pp1a and pp1ab
YP_009725298.1	638	6	nsp2		produced by both pp1a and pp1ab
YP_009725299.1	1945	753	nsp3		former nsp1; conserved domains are: N-terminal
YP_009725300.1	500	9	nsp4		nsp4B_TM; contains transmembrane domain 2 (TM2);
YP_009725301.1	306	747	3C-like proteinase		nsp5A_3CLpro and nsp5B_3CLpro; main proteinase
YP_009725302.1	290	0	nsp6		nsp6_TM; putative transmembrane domain; produced by
YP_009725303.1	83	53	nsp7		produced by both pp1a and pp1ab
YP_009725304.1	198	71	nsp8		produced by both pp1a and pp1ab
YP_009725305.1	113	41	nsp9		ssRNA-binding protein; produced by both pp1a and
YP_009725306.1	139	122	nsp10		nsp10_CysHis; formerly known as growth-factor-like
	932	27	RNA-dependent RNA polymerase		nsp12; NiRAN and RdRp; produced by pp1ab only

YP_009725307.1					
YP_009725308.1	601	133	helicase		nsp13_ZBD, nsp13_TB, and nsp_HEL1core; zinc-binding
YP_009725309.1	527	21	3'-to-5' exonuclease		nsp14A2_ExoN and nsp14B_NMT; produced by pp1ab
YP_009725310.1	346	137	endoRNAse		nsp15-A1 and nsp15B-NendoU; produced by pp1ab only
YP_009725311.1	298	46	2'-O-ribose methyltransferase		nsp16_OMT; 2'-o-MT; produced by pp1ab only
YP_009725295.1	4405	1500	nsp11	orf1ab	pp1a
YP_009725312.1	13	0	nsp11		produced by pp1a only
YP_009724390.1	1273	1473	surface glycoprotein	S	structural protein; spike protein
YP_009724391.1	275	4	ORF3a protein	ORF3a	
YP_009724392.1	75	11	envelope protein	E	ORF4; structural protein; E protein
YP_009724393.1	222	0	membrane glycoprotein	M	ORF5; structural protein
YP_009724394.1	61	0	ORF6 protein	ORF6	
YP_009724395.1	121	4	ORF7a protein	ORF7a	
YP_009725318.1	43	0	ORF7b	ORF7b	
YP_009724396.1	121	4	ORF8 protein	ORF8	
YP_009724397.2	419	140	nucleocapsid phosphoprotein	N	ORF9; structural protein

YP_009725255.1	38	0	ORF10 protein	ORF10	

Reported Efficient Drugs (23 compounds)

- 3D bound protein structures with compounds reported in COVID-19 drug development and COVID-19 drug repurposing research are summarized in a following table.
- We provide analyses "Searching Contact Proteins with Query Compound" of HOMCOS for each compound. Bound protein 3D structures with the compound and its analogues are summarized.

KEGG_ID	structure	NAME	OTHER NAME	Number of identical compounds	Number of similar compounds (tanimoto=0.5)	EFFICACY
D01703	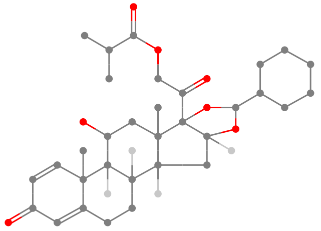	D01703 Ciclesonide (JAN/USAN/INN); Alvesco (TN); Omnaris (TN); Zetonna (TN)	Alvesco (TN); Omnaris (TN); Zetonna (TN)	0	10	Antiasthmatic, Anti-inflammatory, Glucocorticoid receptor agonist
D01425	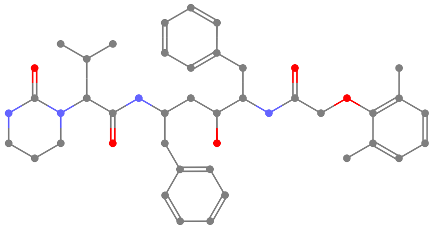	D01425 Lopinavir (JAN/USP/INN)		1	36	Antiviral, HIV protease inhibitor
D00427	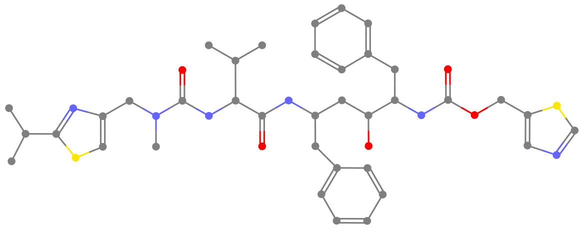	D00427 Ritonavir (JAN/USP/INN); Norvir (TN)	Norvir (TN)	1	31	Antiviral, HIV protease inhibitor
		D09537 Favipiravir	Avigan (TN)	0	62	Antiviral, RNA

D09537	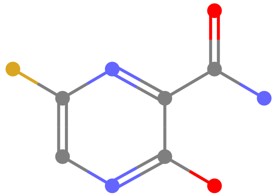	(JAN/USAN/INN); Avigan (TN)				replicase inhibitor
D09537_1	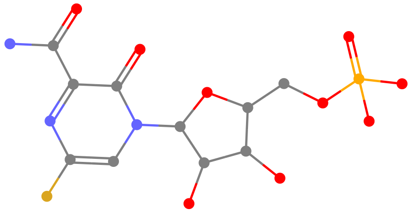	D09537_1 Favipiravir-MTP; Active form of Favipiravir;	Active form of Favipiravir;	1	150	Antiviral, RNA replicase inhibitor
D09537_2	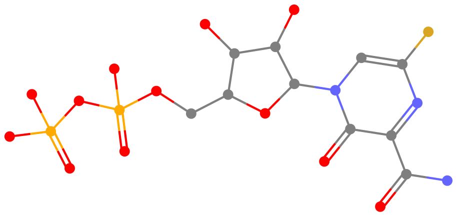	D09537_2 Favipiravir-DTP; Active form of Favipiravir;	Active form of Favipiravir;	0	138	Antiviral, RNA replicase inhibitor
D09537_3	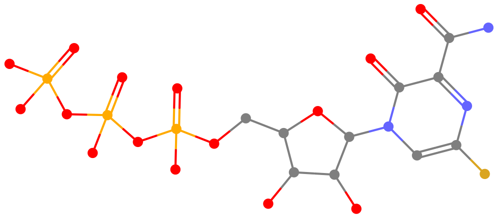	D09537_3 Favipiravir-RTP; Active form of Favipiravir;	Active form of Favipiravir;	1	150	Antiviral, RNA replicase inhibitor
D11472	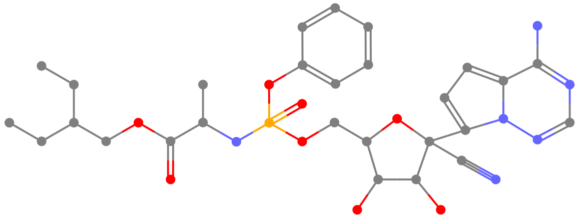	D11472 Remdesivir (JAN/USAN); Veklury (TN)	Veklury (TN)	0	2	Antiviral, RNA polymerase inhibitor
D11472_1		D11472_1 Remdesivir-		1	149	Antiviral

	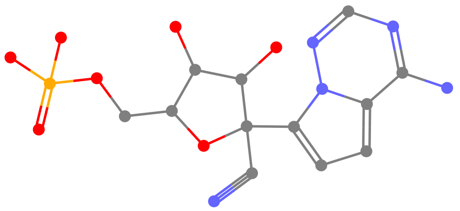	monophosphate (USAN				
D11472_2	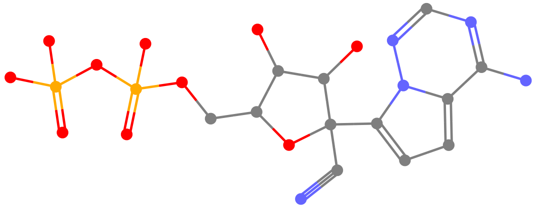	D11472_2 Remdesivir-diphosphate (USAN		0	132	Antiviral
D11472_3	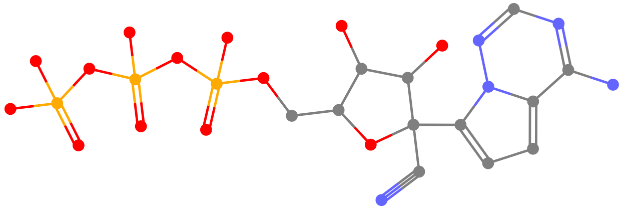	D11472_3 Remdesivir-triphosphate (USAN		0	149	Antiviral
D02366	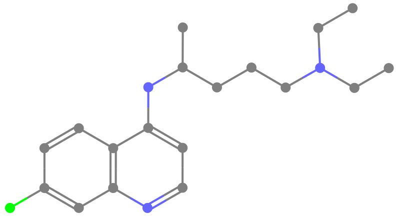	D02366 Chloroquine (USP/INN)		2	31	Antimalarial, Amebicide
D08050	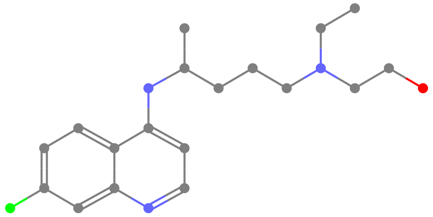	D08050 Hydroxychloroquine (INN); Polirreumin (TN)	Polirreumin (TN)	0	23	Antimalarial
D01670		D01670 Nafamostat	Nafamostat mesilate	0	30	Anticoagulant, Antifibrinolytic,

	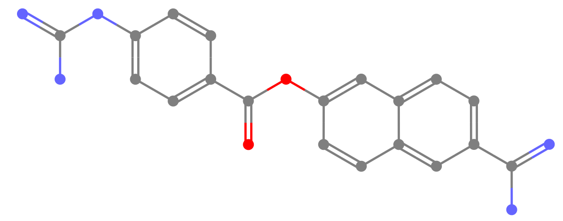	mesylate (USAN); Nafamostat mesilate (JP18); Ronastat (TN)	(JP18); Ronastat (TN)			Serine protease inhibitor
D00429	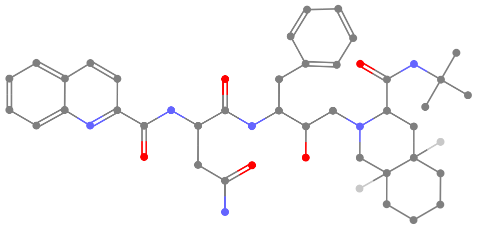	D00429 Saquinavir (JAN/USP/INN); Fortovase (TN)	Fortovase (TN)	1	10	Antiviral, HIV protease inhibitor
D01035	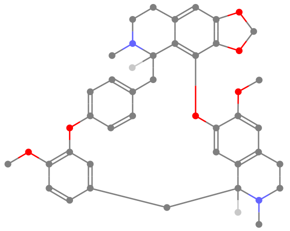	D01035 Cepharanthine (JAN); Cepharanthine (TN)	Cepharanthine (TN)	0	0	Antiallergic, Blood circulation promotor
D00804	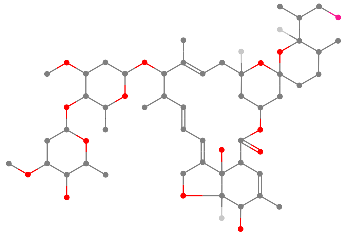	D00804 Ivermectin (JAN/USP/INN); Sklice (TN); Soolantra (TN); Stromectol (TN)	Sklice (TN); Soolantra (TN); Stromectol (TN)	0	1	Antiparasitic
D00292	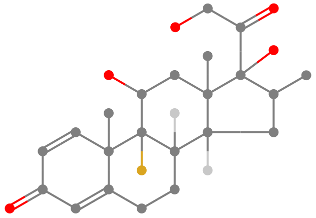	D00292 Dexamethasone (JP18/USP/INN); Decadron (TN); Maxidex (TN)	Decadron (TN); Maxidex (TN)	1	106	Anti-inflammatory, Antipruritic, Glucocorticoid receptor agonist
D07486		D07486 Azithromycin	Azasite (TN); Azithromycin	1	23	Antibacterial, Protein biosynthesis inhibitor

	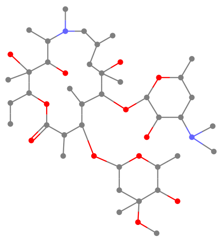	(INN); Azasite (TN); Azithromycin (TN)	(TN)			
[D08897](D08897)	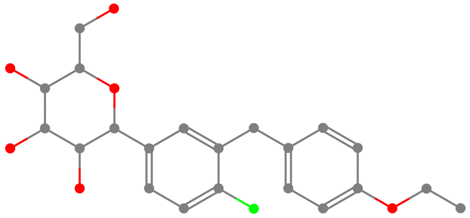	[D08897](D08897) Dapagliflozin (USAN/INN); Forxiga (TN)	Forxiga (TN)	0	33	Antidiabetic, SGLT-2 inhibitor
[D00570](D00570)	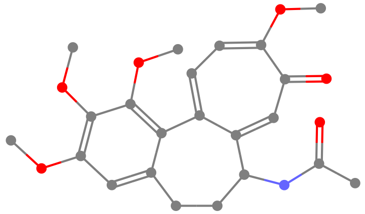	[D00570](D00570) Colchicine (JP18/USP); Colchicine (TN)	Colchicine (TN)	1	7	Gout suppressant, Leukocyte (neutrophil) migration inhibitor, Tubulin polymerization inhibitor
[D07606](D07606)	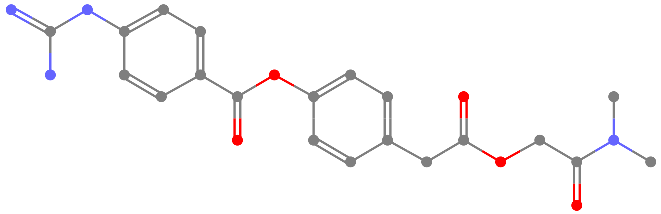	[D07606](D07606) Camostat (INN)		0	14	Anti-inflammatory, Serine protease inhibitor
[D10308](D10308)	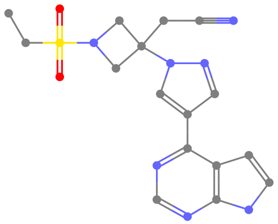	[D10308](D10308) Baricitinib (JAN/USAN/INN); Olumiant (TN)	Olumiant (TN)	1	6	Anti-inflammatory, Antirheumatic, Immunosuppressant, Janus kinase (JAK) inhibitor

---

BLAST is employed for the amino acids sequence search, KCOMBU is used for the chemical structure search.

HOMCOS is developed and maintained by Protein Data Bank Japan, IPR, Osaka University. It is now supported by the project "Platform for Drug Discovery, Informatics, and Structural Life Science" by MEXT, Japan.

References

- Kawabata T. (2016) HOMCOS: an update server to search and model complex 3D structures. *J.Struct.Funct.Genomics*. **17**,83-99. [PubMed] [Publisher]
- Fukuhara N., Kawabata T. (2008) HOMCOS: a server to predict interacting protein pairs and interacting sites by homology modeling of complex structures. *Nucleic Acids Res.*, **36**, W185-W189. [PubMed]

Comments and Questions to: **kawabata@prf.or.jp**
