# Supplementary material for: Protein Data Bank Japan: Celebrating our 20th anniversary during a global pandemic as the Asian hub of three dimensional macromolecular structural data
Source: Protein Sci. 2021 Oct 27;31(1):173–86. doi: 10.1002/pro.4211 (PMC8740847; doi:10.1002/pro.4211)
Supplement: Supplementary file 2 — Appendix S2: Supporting Information [file PRO-31-173-s002.pdf]

Supplementary Information

TITLE: Protein Data Bank Japan: Celebrating our 20th anniversary during a global pandemic as the Asian hub of 3D macromolecular structural data

AUTHORS: Gert-Jan Bekker[1], Masashi Yokochi[1], Hirofumi Suzuki[2], Yasuyo Ikegawa[1], Takeshi Iwata[1], Takahiro Kudo[1], Kei Yura[2], Toshimichi Fujiwara[1], Takeshi Kawabata[3,4], Genji Kurisu[1]

AFFILIATIONS:

1. Institute for Protein Research, Osaka University, 3-2, Yamadaoka, Suita, Osaka 565-0871, Japan
2. School of Advanced Science and Engineering, Waseda University, Shinjuku, Tokyo 169-8555, Japan
3. Protein Research Foundation
4. Graduate School of Frontier Biosciences, Osaka University, 1-3, Yamadaoka, Suita, Osaka, 565-0871, Japan,

Table of Contents

**Section S1**	Generation of 3D conformations of KEGG compounds for HOMCOS' template-based docking	3
**Section S2**	Description of protein sequences and chemical compounds used by the coronavirus webpage on the HOMCOS server	3
**Table S1**	Generation statistics of 3D conformations for chiral compounds for KEGG_COMPOUND	4
**Table S2**	Generation statistics of 3D conformation for chiral compounds for KEGG_DRUG	4
**Table S3**	Statistics of the analysis of chemical similarity between KEGG_COMPOUND against PDB Chemical Components Dictionary by dkcombu	5
**Table S4**	Statistics of the analysis of chemical similarity between KEGG_DRUG against PDB Chemical Components Dictionary by dkcombu	5
**Figure S1**	Overview of different tools and visualizations of 3D structures using Molmil	6
**Figure S2**	New interface of Promode Elastic	8
**Figure S3**	Example of the protein analysis by the webpage for the coronavirus part of the HOMCOS server	10
**Figure S4**	Example of the compound analysis by the webpage for the coronavirus part of the HOMCOS server	12

Supplementary Methods

Section S1: Generation of 3D conformations of KEGG compounds for HOMCOS' template-based docking

Although template-based 3D docking of chemical compounds is useful, the proper initial 3D conformation of the target compound is necessary. To cope with such a situation, we generate 3D conformations of small compounds in the KEGG database,[1] as these compounds have a rich curation describing their reactions, networks, and target proteins. After downloading the 2D structure files in *mol* format from KEGG_COMPOUND (a collection of small molecules relevant to biosystems) and KEGG_DRUG (approved drugs in Japan, USA and Europe), we use Open Babel 2.4.1[2] and the fkcombu[3] program to build the 3D conformations. As building 3D conformations from 2D structures is not a trivial task for compounds with many chiral atoms, we first generate the initial 3D conformations using the babel program with the `--gen3d` option starting from the mol files obtained from KEGG. However, some of generated conformations do not satisfy the chiral information as described in the KEGG mol files. For those failed compounds, we iterate through the following three steps until the correct conformation is achieved. (1) The conformations of chiral-miss-match atoms are modified by the program fkcombu, using the operations pyramid inversion, reflection, and folding hex-ring. (2) Conformations are then relaxed using the program obminimize and (3) checking the chiral atoms of the modified conformations against the KEGG mol file using the program pkcombu. The statistics of the generated chemical compounds are shown in Table S1 and Table S2. Most of the generated 3D conformations of chiral compounds have a consistent chirality with those described in the mol file obtained from KEGG, with a success rate of 10404/10484 for KEGG_COMPOUND, and 3513/3519 for KEGG_DRUG.

Section S2: Description of protein sequences and chemical compounds used by the coronavirus webpage on the HOMCOS server

For the SARS-CoV-2 webpage of HOMCOS, the sequences and compounds related to SARS-CoV-2 are collected as follows. The amino acid sequences were taken from the UniProt FTP site (ftp.uniprot.org/pub/databases/uniprot/pre_release). Here, the UniProt pre-release dataset contains not only sequences of SARS-CoV-2 (13 proteins), but also SARS-CoV (15 proteins) and human proteins (16 proteins) related to the virus, such as ACE2_HUMAN and FURIN_HUMAN. In addition to the UniProt data, we've also added 28 proteins corresponding to the GenPept identifier NC_045512, where 15 proteins in the GenPept entry (including 3C-like proteinase) of the polyprotein pp1ab (R1AB_SARS2) were separated into 15 entries.

The compounds part of KEGG_DRUG (https://www.genome.jp/kegg/drug/), which stores approved drugs in Japan, USA and Europe, are now also stored on the HOMCOS server. Among the

compounds in KEGG_DRUG, 23 potential repurposing drugs, including approved drugs as listed on Wikipedia (https://en.wikipedia.org/wiki/COVID-19_drug_repurposing_research). In addition, the prodrugs of favipiravir and remdesivir in their phosphate forms have also added to the compound list.

REFERENCES

1. Kanehisa M, Furumichi M, Sato Y, Ishiguro-Watanabe M, Tanabe M (2021) KEGG: integrating viruses and cellular organisms. Nucleic Acids Res. 49:D545–D551.

2. O'Boyle NM, Banck M, James CA, Morley C, Vandermeersch T, Hutchison GR (2011) Open Babel: An open chemical toolbox. J. Cheminform. 3:33.

3. Kawabata T (2011) Build-Up Algorithm for Atomic Correspondence between Chemical Structures. J. Chem. Inf. Model. 51:1775–1787.

Supplementary Tables

Table S1. Generation statistics of 3D conformations for chiral compounds for KEGG_COMPOUND [a]

	Number of Compounds
Total	18174
with chiral atoms	10484
with chiral atoms successfully built by OpenBabel babel	7137
with chiral atoms unsuccessfully built by OpenBabel babel	3347
with chiral atoms successfully built by *fkcombu* and OpenBabel *obminimize*	10404
with chiral atoms unsuccessfully built by *fkcombu* and OpenBabel *obminimize*	80

a)

Table S2. Generation statistics of 3D conformation for chiral compounds for KEGG_DRUG [a]

	Number of Compounds
Total	8686
with chiral atoms	3519
with chiral atoms successfully built by OpenBabel babel	2529
with chiral atoms unsuccessfully built by OpenBabel babel	990
with chiral atoms successfully built by fkcombu and OpenBabel *obminimize*	3513
with chiral atoms unsuccessfully built by *fkcombu* and OpenBabel *obminimize*	6

a) version 2021/08/23

Table S3. Statistics of the analysis of chemical similarity between KEGG_COMPOUND [a] against PDB Chemical Components Dictionary [b] by dkcombu [c]

	Number of Compounds
Total	18174 (100.0 %)
identical compounds (Tanimoto index = 1.0))	4865 (26.8 %)
similar compounds (Tanimoto index > 0.7 and < 1.0)	8560 (47.1 %)
similar compounds (Tanimoto index > 0.5 and < 1.0)	12067 (66.4 %)
similar compounds (Tanimoto index <= 0.5)	1242 (6.8 %)

[a)] Version 2021/08/23

[b)] Version 2021/08/11

[c)] The structure comparison was done using TD-MCS with θ=1.

Table S4. Statistics of the analysis of chemical similarity between KEGG_DRUG [a] against PDB Chemical Components Dictionary [b] by dkcombu [c]

	Number of Compounds
Total	8686 (100.0%)
identical compounds (Tanimoto index = 1.0)	2548 (29.3 %)
similar compounds (Tanimoto index > 0.7 and < 1.0)	3306 (38.1 %)
similar compounds (Tanimoto index > 0.5 and < 1.0)	5454 (62.8 %)
similar compounds (Tanimoto index <= 0.5)	570 (6.7 %)

[a)] Version 2021/08/23

[b)] Version 2021/08/11

[c)] The structure comparison was done using TD-MCS with θ=1.

Supplementary Figures

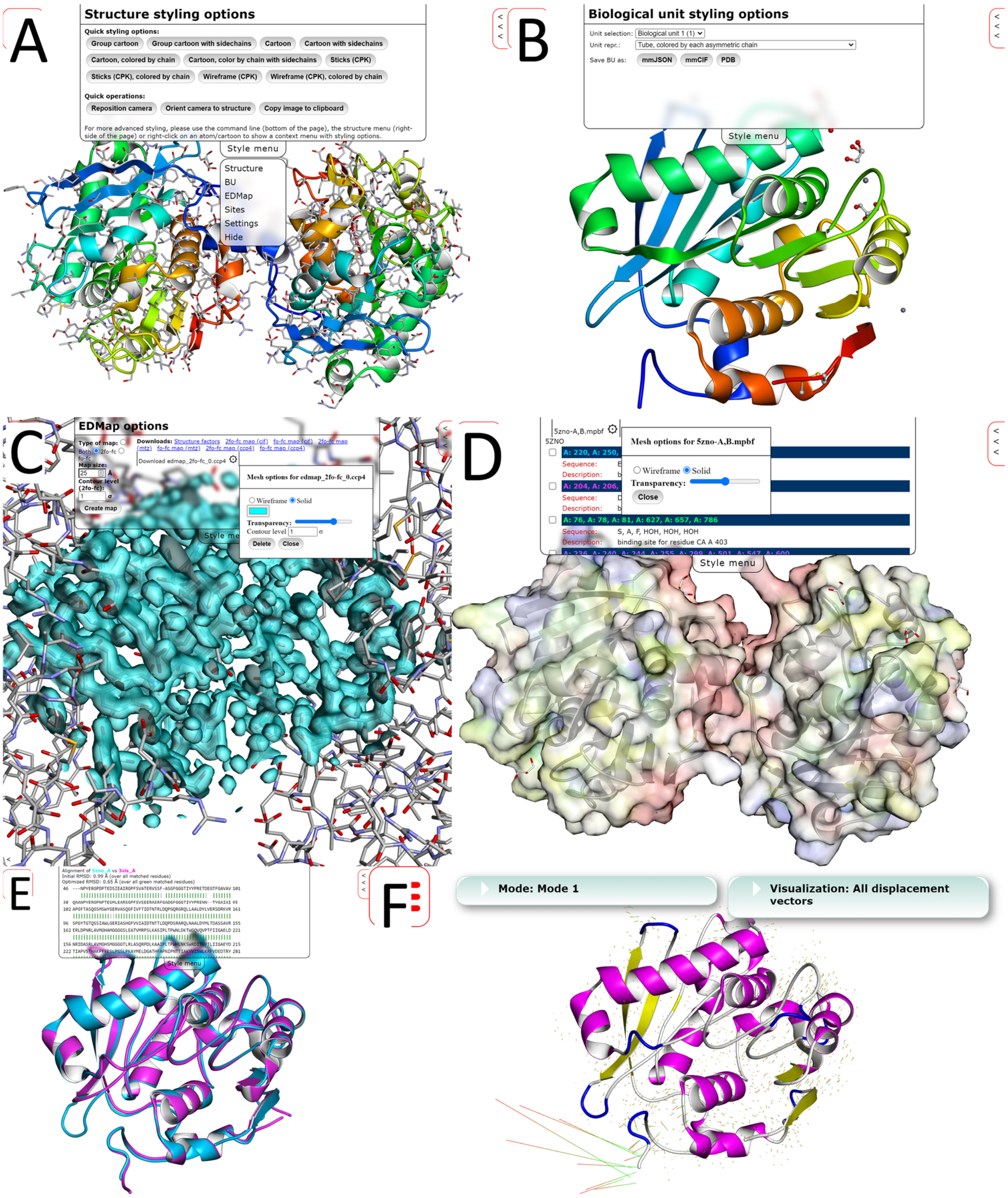

Figure S1. **Overview of different tools and visualizations of 3D structures using Molmil**. (A) Basic visualization (for the Asymmetric Unit, AU), where different styles for the structure can be selected within the panel. (B) Visualization options for the Biological Unit (BU). The BU type and the representation of the BU (for additional chains not part of the AU) can be selected here. (C) For entries that include structure factor data, electron density maps are also viewable using Molmil via our

EDMap service. A map for a localized area can be generated by clicking on an atom in the canvas and then clicking on the “Create map” button. To change the representation of a map, users can click on the options icon to show a menu (“Mesh options for edmap_2fo-fc_0.ccp4”), where the representation, color, transparency and contour level can be modified (the map can also be deleted). (D) List of available sites (either from the PDB metadata or our own pdbjplus data), where clicking on an item shows the residues, and double clicking jumps to these residues. If an eF-site entry was selected from the PDB entry page, a surface representation for the corresponding eF-site entry is also shown, where its styling can be modified in the same manner as for a map with our EDMap service. (E) Results found by our Sequence Navigator/Neighbor service can also be visualized by Molmil. Molmil performs the sequence alignment of the provided chains and then shows the alignment in addition to the superposed molecules and their RMSD. (F) Representation of displacement vectors by Molmil for our Promode Elastic service (not part of the new style system, but is directly integrated onto the Promode Elastic entry page, see also Figure S2). For all subfigures, the data for the entry with PDB ID 5ZNO (https://pdbj.org/mine/summary/5zno) was used to produce the images.

5zno PDBj Mine

5zno

Download raw data: 5zno-results.tgz

Copyright © WASEDA Univ,Japan. All rights reserved.

Interactive 3D visualization

Mode: Mode 1

Visualization: All displacement vectors

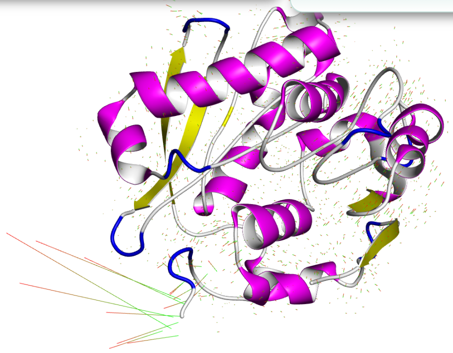

* To zoom using the mousewheel, please hold the CTRL button.

Statistics

Statistics: Fluctuation of atoms

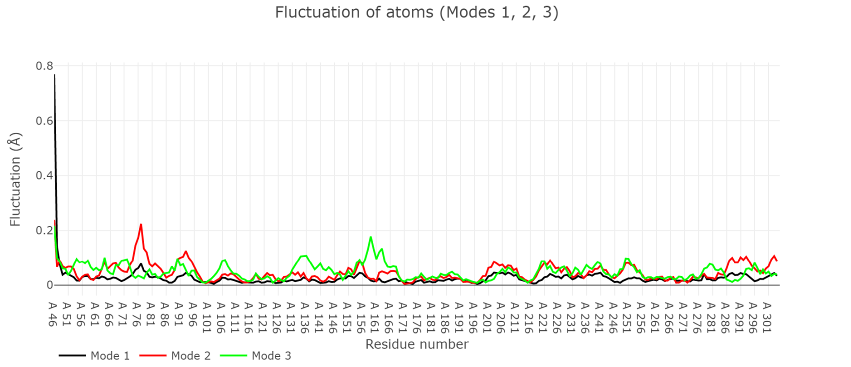

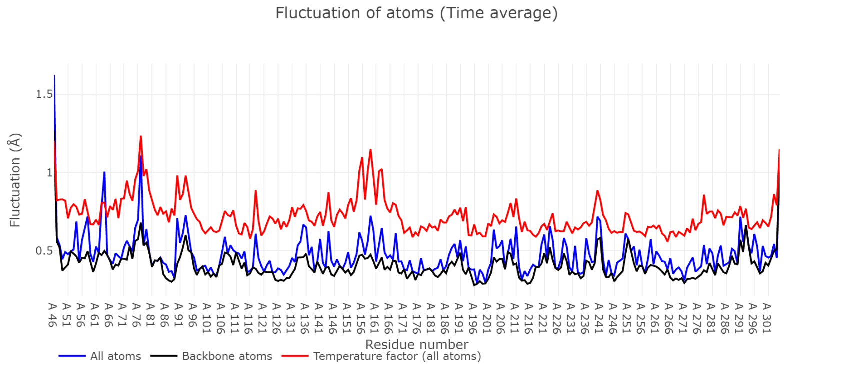

Header

Calculation note

Figure S2. **New interface of Promode Elastic**. The top panel on the entry page shows an interactive 3D visualization of the displacement vectors or an animation using Molmil, which can be selected using the menus. The statistics panel directly below the 3D structure shows various statistics in an interactive manner (the graphs can be zoomed in, where hovering the mouse over the graph shows the corresponding raw data values). The data for the entry with PDB ID 5ZNO was used to produce the images, available at https://pdbj.org/promode-elastic/5zno.

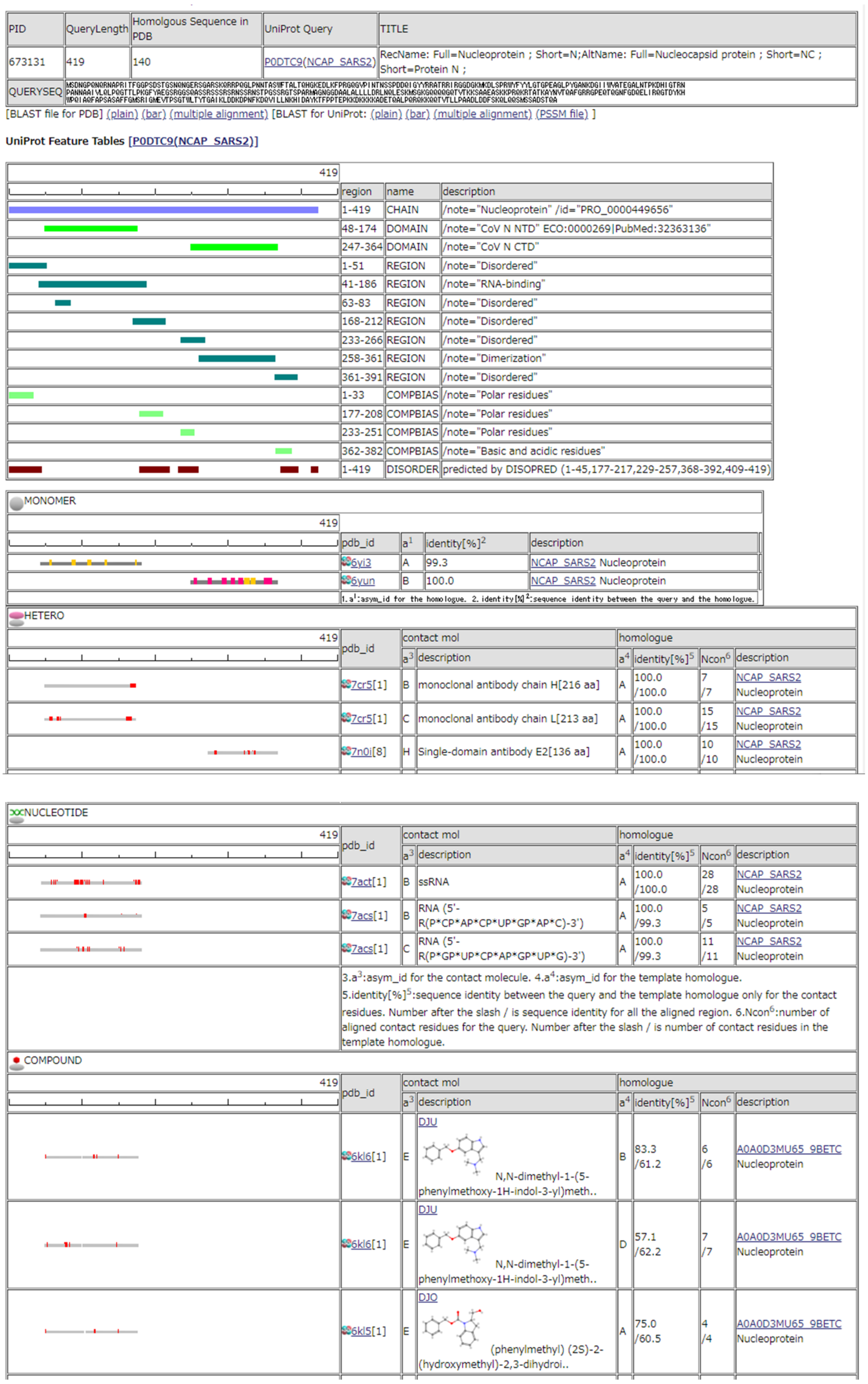

Figure S3. **Example of the protein analysis by the webpage for the coronavirus part of the HOMCOS server.** The shown analysis is for Protein N (NCAP_SARS2) of SARS-CoV-2. The first part is the annotation from UniProt, with predicted disordered regions. The "MONOMER" section lists representative homologous 3D structures, while the "HETERO" section indicates interaction sites of homologous hetero complexes. The "NUCLEOTIDE" section shows interaction sites of

homologous nucleotide complexes, while the “COMPOUND” section shows interactions sites of compound-protein complexes. Clicking the “3D” icon visualizes the molecules using a 3D molecular viewer and provides template-based modeling options.

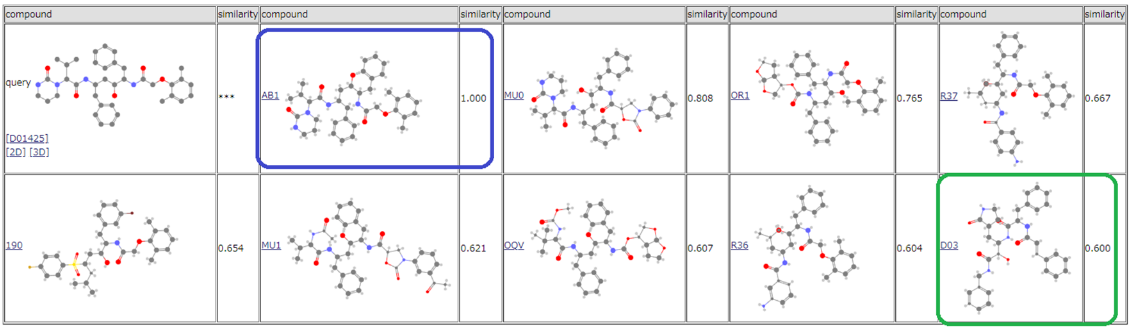

Proteins contacting with similar compounds to the query

Nrank	Protein	Nresidue	pdb_id+asym_id
1	Q5UEA2_9ENTO Genome polyprotein	180	[D03]:5nfs_A_B
2	POL_FIVPE FIV protease	112	[AB1]:3ogq_A_C [AB1]:3ogq_B_C
3	R1AB_CVHSA SARS coronavirus main protease	306	[D03]:5n19_A_B [D03]:5n19_A_B
4	R1AB_CVHNL 3C-like proteinase	301	[D03]:6fv2_A_E [D03]:6fv2_B_G [D03]:6fv2_C_I
5	Q9QM22_9HIV1 protease	99	[AB1]:1rv7_A_C [AB1]:1rv7_B_C [AB1]:4l1a_A_C [AB1]:4l1a_B_C
6	A0F7J4_9HIV1 PROTEASE RETROPEPSIN	99	[AB1]:2rkf_A_D [AB1]:2rkf_B_D [AB1]:2rkg_A_D [AB1]:2rkg_B_D
7	PLM2_PLAFA Plasmepsin 2	329	[R37]:1lf2_A_B [R36]:1lee_A_B [R36]:1lee_A_B [R36]:1lee_A_B [R36]:1lee_A_B [R36]:1lee_A_B [R36]:1lee_A_B [R36]:1lee_A_B [R36]:1lee_A_B [R36]:1lee_A_B [R36]:1lee_A_B [R36]:1lee_A_B [R36]:1lee_A_B
8	Q903J0_9HIV1 protease	99	[AB1]:1mui_A_C [AB1]:1mui_B_C [AB1]:2o4s_A_D [AB1]:2o4s_B_D [AB1]:2q5k_A_F [AB1]:2q5k_B_F [AB1]:2qhc_A_E [AB1]:2qhc_B_E [AB1]:2z54_A_D [AB1]:2z54_B_D [AB1]:6dj1_A_E [AB1]:6dj1_B_E [AB1]:6dj2_A_E [AB1]:6dj2_B_E [AB1]:6pjb_A_K [AB1]:6pjb_B_K [MU0]:2q55_A_E [MU0]:2q55_B_E [OR1]:6pjc_A_G [OR1]:6pjc_B_G [OR1]:6pjc_C_J [OR1]:6pjc_D_J [190]:1xl5_A_F [190]:1xl5_B_F [MU1]:2q54_A_G [MU1]:2q54_B_G [OQV]:6pjm_A_E [OQV]:6pjm_B_E

Figure S4. **Example of the compound analysis by the webpage for the coronavirus part of the HOMCOS server.** Here, the analysis of Lopinavir (D01425), an approved drug for HIV, where research has shown evidence of activity against SARS and MERS. An identical compound to Lopinavir has been registered in the PDB with a comp_id of "AB1" (shown in the blue box), however, this compound does not exist within the PDB in complex with any proteins from coronaviruses. On the other hand, while the compound "D03" (shown in the green box) has a weak similarity (Tanimoto index = 0.6) to Lopinavir, a complex structure of D03 with the main protease (3C-like protease) of a coronavirus (not SARS-CoV-2) does exist (shown in the red box). Using this structure as template, we can model the complex 3D structure of 3C-like protease of SARS-CoV-2 and Lopinavir, as shown in Figure 4.
